# Exposure to open defecation can account for the Indian enigma of child height^☆^

**DOI:** 10.1016/j.jdeveco.2018.08.003

**Published:** 2020-09

**Authors:** Dean Spears

**Affiliations:** a Department of Economics and Population Research Centre, University of Texas at Austin, Austin, TX, USA; b Economics and Planning Unit, Indian Statistical Institute, Delhi, India; c IZA, Germany; d r.i.c.e., USA

## Abstract

Physical height is an important measure of human capital. However, differences in average height across developing countries are poorly explained by economic differences. Children in India are shorter than poorer children in Africa, a widely studied puzzle called “the Asian enigma.” This paper proposes and quantitatively investigates the hypothesis that differences in sanitation — and especially in the population density of open defecation — can statistically account for an important component of the Asian enigma, India's gap relative to sub-Saharan Africa. The paper's main result computes a demographic projection of the increase in the average height of Indian children, if they were counterfactually exposed to sub-Saharan African sanitation, using a non-parametric reweighting method. India's projected increase in mean height is at least as large as the gap. The analysis also critically reviews evidence from recent estimates in the literature. Two possible mechanisms are effects on children and on their mothers.

## Highlights

• Indian children are shorter than African children, a puzzle called the Asian enigma.
• We project a counterfactual increase in the average height of Indian children.
• The counterfactual matches sub-Saharan African exposure to open defecation density.
• India's projected increase in mean height is at least as large as the gap.

## 1 Introduction

Physical height is of wide interest to economists (Steckel, 2009), in large part because it is a strong and observable correlate of human capital and health and is a predictor of economic productivity (Case and Paxson, 2008). Despite careful attention and significant research about height, human capital, and economic well-being, an important puzzle persists: international differences in height across developing countries are not well explained by differences in economic wealth (Deaton, 2007; Jayachandran and Pande, 2017).

**Fig. 1:**
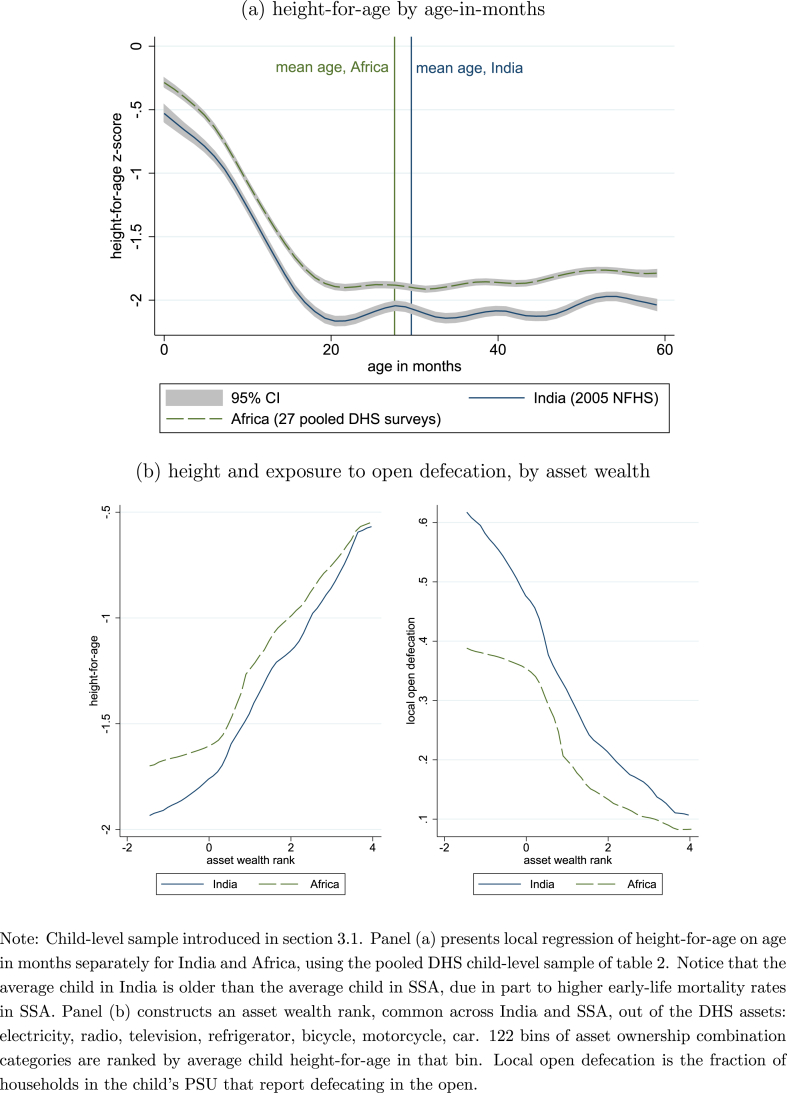
The enigma: Height in India and Africa.

In particular, people in India are shorter on average than people in sub-Saharan Africa, despite the fact that Indians are also richer on average (Fig. 1). This enduring and important paradox in the literature has been called the “Asian enigma” (Ramalingaswami et al., 1996). India's height deficit relative to Africa is large: it is about two-fifths as large as the average rural-urban height gap in India, and almost one-quarter as large as the average difference in India between children of literate and illiterate mothers. This defecit impacts many people because 19% of births occur in India.

Sanitation, when poor, can be a key determinant of human capital in developing countries. This is because germs from feces cause diarrhea and other diseases, which can consume energy and harm the overall nutrition of growing children and of the mothers who nurture them in pregnancy and early life. Medical research indicates that chronic environmental exposure to fecal germs could be an important cause of growth deficits. This paper documents a large and robust gradient between exposure to open defecation and child height. As we show, sanitation can explain international variation in child height that income and other dimensions of development cannot. Our main conclusion is that open defecation can statistically account for much or all of the average height-for-age difference between India and Africa, in the sense that children in India are projected to be taller by at least as much as the gap, if counterfactually exposed to the African distribution of open defecation density.

Over one billion people worldwide defecate in the open (meaning without using any toilet or latrine), most of these in India (Coffey and Spears, 2017). For reasons related to the history and endurance of the caste system, sanitation coverage is exceptionally poor in India, a densely populated country where most people^1^ defecate in the open. This is a much larger prevalence of open defecation than in other countries with similar economic status. Perhaps surprisingly in light of how much open defecation there is, and how different sanitation in India is from sanitation elsewhere, sanitation received little prior attention in economists' wide-ranging investigations of the puzzle of Indian malnutrition and stunting (*e.g.* Deaton, 2007; Tarozzi, 2008; Jensen, 2012; Panagariya, 2013).

This paper makes several contributions to the literatures on development economics, human capital, and health. First, it proposes and analyzes a hypothesis to resolve an important puzzle which has attracted enduring attention in the literature. Second, this paper contributes to a literature in economics focused on early-life human capital accumulation (Cunha et al., 2010; Gertler et al., 2014). In this literature, height is a key variable, especially in developing countries where health is a particularly important dimension of children's human capital (Maluccio et al., 2009; Currie and Vogl, 2013; Hoddinott et al., 2013). Third, by advancing evidence on the importance of sanitation and open defecation in developing countries, especially in India, this paper contributes to the economic case that reducing open defecation – a classic public bad with significant externalities – is a policy priority.

Section 2 provides background. It introduces the Asian enigma, summarizes evidence from the economic and biological literature that an important effect of sanitation on child height is plausible, and reviews evidence on why open defecation is so uniquely persistent in India. For open defecation to explain the India-SSA height gap, two things must be true: first, the association between height and open defecation in India must be quantitatively large enough to account for the gap, and second, this association must plausibly reflect a causal effect of exposure to poor sanitation, rather than other coincidentally correlated variables. The demographic reweighting method at the heart of this paper's contribution focuses on the first requirement; to support the second, this paper considers and integrates other evidence, including estimates in the literature that have emerged since the initial working paper version of this analysis.

Section 3 introduces the empirical strategy. Section 4 presents the main result of this paper: a demographic projection of the consequences for average child height if Indian children were exposed to the distribution of open defecation density to which African children are exposed. Then, section 5 reports two extensions: a linear regression that permits a richer set of controls than the non-parametric projection, and suggestive evidence of a mechanism through maternal nutrition, which would have effects, in turn, on fetal and early-life growth. Finally, section 6 integrates evidence from recent estimates in the literature of effects of open defecation on child height. This section computes a meta-estimate that is quantitatively closely consistent with the effect size necessary for open defecation to be able to account for the India-Africa height gap.

## 2 Background

### 2.1 The Asian enigma: the gap to be explained

Many health outcomes in India are much worse than would be predicted in international comparison, based on India's GDP per capita and other measures of economic performance (Drèze and Sen, 2013). India's infant mortality rate is about one-third higher than those of Bangladesh and Nepal, although these are poorer countries. Anemia, too, is poorly explained by income (Alderman and Linnemayr, 2009) and is unusually common in India (Kassebaum et al., 2014) despite India lacking a high malaria burden similar to sub-Saharan Africa's (Coffey et al. 2017a,b; 2018). Over 40% of women in India are underweight when they become pregnant (Coffey, 2015), and even a quarter of working age adult men are underweight, a fact suggesting shared environmental causes in addition to the social forces that deprive young women.

Among these poor health outcomes in India, child height has received particular attention in the economics literature. Fig. 1 depicts the gap to be explained: children in India are shorter than children in SSA at each age and each level of household asset wealth. As Deaton (2013) writes about the “startling … enormous inequality of average heights around the world,” “the fact that South Asians are so short is perhaps the most informative part of the whole picture.” Ramalingaswami et al. (1996) named this phenomenon an Asian enigma, but it is principally an Indian enigma (Headey et al., 2015). As Ghosh et al. (2014) showed, for example, children in Bangladesh only appear shorter than children in West Bengal because they are poorer, on average; at the same level of asset wealth, children in West Bengal are statistically significantly shorter than their Bangladeshi neighbors.^2^ Children in China – where, among other differences, open defecation is now rare even in rural places – are taller than children in sub-Saharan Africa and much taller than children in India, on average.

The principal goal of this paper is to quantitatively assess the degree to which sanitation can account for the Asian enigma. This depends on the size of the gap in child height, the size of the difference in exposure to poor sanitation, and the size of the relevant effect of sanitation on height. To quantify the size of the Asian enigma gap, we use child-level data from Demographic and Health Surveys (DHS). Our sample follows Jayachandran and Pande's (2017) construction of a sub-Saharan African (hereafter SSA) sample of DHS rounds, which we detail in section 4. With these data, we estimate a height gap of 0.146 height-for-age standard deviations (clustered 95% CI: 0.115 to 0.176) by regressing height-for-age on only an indicator for living in India rather than SSA.^3^ Throughout this paper, when we refer to “height” or the height gap, we mean height-for-age.

Consider the following linear approximation to the task of quantitatively accounting for the Asian enigma:

(1) $$\text{required}\;\hat{\beta }\approx \frac{\text{India}‐\text{Africa}\;\text{difference}\;\text{in}\;\text{child}\;\text{height}‐\text{for}‐\text{age}}{\text{India}‐\text{Africa}\;\text{difference}\;\text{in}\;\text{exposure}\;\text{to}\;\text{open}\;\text{defecation}}.$$

The numerator is the height gap. To illustrate the denominator, the difference in exposure to open defecation between Indian and African children in the same DHS data is 31.2 percentage points, resulting in a required average, linear effect size of 0.47. Therefore, to the extent that the average effect of local open defecation on child height within India is around 0.5 height-for-age points associated with moving from 100% open defecation in a village to 0%, we would interpret effect estimates as evidence that hypothetically causing India to match SSA open defecation would predict an increase in child height in India approximately as large as the India-Africa gap. Most of this paper, however, implicitly uses a different denominator: the difference between Indian and African children in exposure to open defecation *density* (Hathi et al., 2017).^4^ Our focus on this variable emphasizes the “public bad” nature of poor sanitation, and the fact that Indian children are disadvantaged by being exposed to open defecation in a context where people live near to one another.

### 2.2 Why open defecation may influence child height

Although no prior paper has sought to explain international heterogeneity in height via sanitation, a substantial literature suggests the plausibility of this possibility. Several papers in economics have documented large effects of sanitation-related disease on early-life health (*e.g.* Galiani et al., 2005; Cutler and Miller, 2005; Alsan and Goldin, 2015; Geruso and Spears, 2018). Further econometric research has traced sanitation-related early-life disease through to human capital and economic outcomes (Spears and Lamba, 2016; Baird et al., 2011). For example, Bleakley (2007) showed that eradicating hookworm infection – one of several mechanisms by which poor sanitation impacts health – improved learning and increased incomes in the American South.

More generally, disease is increasingly recognized as a potential determinant of population-level height. The economic history literature has shown a large association between average population-level heights and the disease environment, as reflected in mortality rates (Bozzoli et al., 2009). Hatton (2013), studying the historical increase in European height, concludes that “the most important proximate source of increasing height was the improving disease environment as reflected by the fall in infant mortality.”

There are at least two mechanisms by which exposure to open defecation might reduce population-level average height. One mechanism is through the exposure of children, after they are born. Net nutrition and disease interact (Smith et al., 2013). It has long been documented that diarrhea could cause stunting due to loss of consumed nutrients (*e.g.* Guerrant et al., 1992; Checkley et al., 2008). Most recently documented in detail in the medical literature, but perhaps important, is the possibility of chronic but subclinical “environmental enteric disfunction” (Humphrey, 2009). EED would be caused by repeated fecal contamination which, through an inflammatory response, increases the small intestine's permeability to pathogens while reducing nutrient absorption. Such inflammation could cause height deficits even without necessarily manifesting as diarrhea or otherwise observable illness.^5^

The other possible mechanism is the size and net nutrition of mothers, which could be influenced by near-term exposure to open defecation during pregnancy, or by long-term exposure over the course of the mother's life. Behrman et al. (2009), for example, show that girls who received a nutritional supplement in Guatemala grew up to be mothers who had taller children with greater birth weight – evidence of a long-lived, intergenerational effect of mothers' net nutrition. Population-level data on birth weight is unavailable in many developing countries, including India. However, evidence from developed countries shows that both mothers' pre-pregnancy body mass and weight-gain in pregnancy substantially shape birth weight, especially for underweight women (Institute of Medicine, 1990; Yaktine and Rasmussen, 2009; Han et al., 2011). If maternal nutrition matters for child anthropometry, then it is plausible that it could be influenced by disease, as well as nutritional intake.^6^ Motivated by the hypothesis that “since stunting begins *in utero*, the maternal inflammatory environment may have an important influence on fetal growth,” Prendergast et al. (2014) measured birthweight, child size in infancy, and chemical biomarkers of inflamation and intestinal damage in infants and mothers in Zimbabwe. They found that “birth weight was related to infant IGF-1 [a childhood growth hormone] at birth, which in turn was associated with the inflammatory status of the mother-infant dyad. The infant inflammatory milieu was closely related to the level of maternal inflammation at birth.” This paper further considers evidence for a maternal nutrition mechanism in section 5.2.

Section 6 considers the quantitative implications of some studies in the biomedical, demographic, and economic literatures. It is beyond the scope of this econometric paper to decompose the association between sanitation and height among various biological mechanisms, although diarrhea, chronic intestinal disease, worm and parasite infections, and energy requirements while fighting disease are all well-documented pathways that make an effect of open defecation on child height plausible. Especially in India — where cross-sectional, geographic differences in open defecation have changed only very slowly (Coffey and Spears, 2018c) — a cumulative effect through the health and size of mothers is also plausible.

### 2.3 Why open defecation persists in India — and consequences for studying health effects

In light of this biological science, sanitation has a plausible place among the candidate explanations for poor health outcomes in India because open defecation is so uniquely widespread in India. Most people in the world who defecate in the open live in India. Open defecation rates in India are higher than in many poorer countries: in only a few small countries does a larger fraction of the population defecate in the open than in India.^7^ Finally, population density is high even in rural India, so it is more likely that germs from any quantity of open defecation would be able to cause infection.

A natural question, then, is why open defecation in India remains so common, despite economic growth. The explanation is not poverty: open defecation is less common in many poorer countries; simple latrines are affordable; and even many people in rural India who live in households that own working latrines choose to defecate in the open rather than use them (Coffey et al., 2014; Clasen et al., 2014). Coffey and Spears (2017) present qualitative and quantitative evidence that the causal roots are in casteism, untouchability, and ideas of ritual purity and pollution. In short, a core reason is that many people in rural India are unwilling to use pit latrines because they are concerned about what will happen when the latrine pit fills. Emptying a latrine pit — which is done every few years in other developing countries by household members or people they hire — is associated with the most degrading ritual impurity, and subjectively can only be done by members of the lowest castes.^8^

Spears and Thorat (2017) provide quantitative support for this explanation, using the India Human Development Survey (IHDS). The IHDS asked a novel survey question about whether members of a household practice untouchability, in the sense of enforcing the rules of untouchability in interactions with members of lower-ranking castes. Spears and Thorat show that people living in villages where more of their neighbors report practicing untouchability are more likely to defecate in the open.

One consequence of the social forces behind the slow decline in open defecation in India is that it is difficult for an intervention study to cause a large decline in village-level open defecation in rural India. Yet, the ability to successfully induce a large decline in open defecation is necessary for the first stage — in the sense of an instrumental variables analysis — of any cluster randomized trial to study the effect of open defecation on child height or other outcomes (Spears and Haddad, 2015). Although experiments have generated large enough first stages to learn from in contexts outside of India (Pickering et al., 2015; Gertler et al., 2015), RCTs *in India* have not shown similarly large first stage effects on open defecation behavior (Clasen et al., 2014; Patil et al., 2014; Hammer and Spears, 2016). This is important to this paper's goal of understanding the relationship between height and open defecation in India, because India's high population density gives reason to expect that the effect in India is larger than in other contexts. Another reason that it would be difficult to answer our question with an RCT about reducing open defecation conducted in another country is that increasingly few other countries have much open defecation left at all. In those contexts, an experiment could study a further improvement in sanitation — such as from latrine use to improved toilet use — but that would not be an estimate of the effect of open defection. One hopes this constraint will eventually be overcome as further research develops strategies to rapidly reduce open defecation in Indian villages, allowing statistically powerful intervention studies of its long-term consequences there — although, even then, effects through accumulated maternal exposure may require a long duration of changed exposure to reverse. At least until such techniques are available, there will be uncertainty about the exact size of the effect, and there will remain an important role for population-level observational analysis.

## 3 Empirical strategy

This paper applies a decomposition analysis in the spirit of Oaxaca-Blinder to provide a quantitative answer to our main question: would counterfactually changing Indian children to be exposed to the African distribution of open defecation increase their height by an amount as large as the India-Africa height gap? In particular, we apply the non-parametric reweighting method of DiNardo et al. (1996) to project the average height of Indian children, if they were exposed to the same distribution of open defecation density as African children. This approach has the advantage of matching the full distribution of exposure to open defecation, not only mean differences. It also assumes no functional form. Similar decomposition methods have recently been used in the economics literature to study demographic puzzles by Bhalotra et al. (2010) and by Chen et al. (2016).

### 3.1 Data and summary statistics

Applying this strategy requires an Indian and an African sample of child heights. Any construction of an African sample out of country-level surveys must be somewhat arbitrary. This paper follows the sample constructed in a recent paper by Jayachandran and Pande (2013, 2017), pooling child-level data from India and 27 recent DHS from sub-Saharan Africa.^9^ The Indian sample is India's 2005 DHS.

The key independent variable of this analysis is open defecation density. The log^10^ of open defecation density is computed as

$$\ln\left(local\;open\;defecation\times population\;density+1\right),$$

where *local open defecation* is the computed fraction $\left[0,1\right]$ of households in a child's survey primary sampling unit (PSU) who defecate in the open, and *population density* is matched from census data sources at the level of Indian states and African countries. The open defecation independent variable is, of course, measured at the time of the survey — which means that it best describes the disease environment faced in early life by the youngest children, born at a time closest to the survey.^11^ Any bias introduced by the implicit assumption that open defecation at the time of the survey is similar to open defecation a few years earlier is likely to be small in India, however, because open defecation has changed so slowly (Coffey and Spears, 2017). In particular, Coffey and Spears (2018c) compute that district-level rural open defecation rates in India's 2011 Census and 2015-6 NFHS-4 have a correlation of 0.93, documenting that change was slow over a five-year period (which is the same age range for height measurement) even during a high-profile national sanitation policy.

Table 1 presents summary statistics. The table presents results by age-in-months sub-samples, a practice that will be repeated throughout the results. Height-for-age is well-understood to be correlated with age-in-months according to a common pattern, as in Fig. 1. A focus on younger ages would have the advantage that the open defecation environment recorded at the time of the survey more closely matches the open defecation environment in younger children's infancy, if open defecation has been changing over time. A focus on older ages would have the advantage that age is not mechanically correlated with height-for-age. There is an India-SSA mean height difference in every age group, which suggests, in part, that differences begin *in utero*.

**Table 1:** Sample means by population and age.

	all ages		0–5 months		6–24 months		25–59 months	
	India	SSA	India	SSA	India	SSA	India	SSA
**Dependentvariable**:								
height-for-age *z*-score	−1.824	−1.541	−0.542	−0.310	−1.736	−1.467	−2.063	−1.839
**Independentvariables**:								
open defecation (own)	0.581	0.311	0.554	0.319	0.570	0.305	0.590	0.313
open defecation (PSU)	0.630	0.317	0.660	0.327	0.633	0.313	0.625	0.318
ln(OD density)	4.943	1.893	5.092	1.904	4.947	1.880	4.918	1.899
population density ($\frac{\text{pop}}{{\text{km}}^{2}}$)	598.7	74.0	604.7	72.8	592.8	73.2	600.9	74.6
**Demographics:**								
age in months	29.85	27.87	2.90	2.76	14.80	14.57	41.93	41.41
sibsize at survey	2.93	3.68	2.62	3.40	2.60	3.41	3.15	3.90
birth order	2.75	3.75	2.73	3.73	2.65	3.72	2.80	3.78
boy	0.523	0.502	0.492	0.504	0.524	0.500	0.528	0.502
first-born boy	0.152	0.101	0.148	0.105	0.163	0.103	0.147	0.100
last-born at survey	0.690	0.609	0.942	0.846	0.909	0.825	0.536	0.423
**Socio-economic status:**								
assets (of 5)	1.907	1.513	1.710	1.460	1.921	1.498	1.929	1.533
urban	0.247	0.253	0.222	0.245	0.245	0.252	0.252	0.255
mother literate	0.507	0.600	0.507	0.596	0.530	0.614	0.496	0.592
father no education	0.281	0.321	0.273	0.330	0.265	0.309	0.291	0.326

*Note:* Unlike other results in this paper intended to document relationships, here sampling weights are used.

Table 1 reveals several important patterns. One is that the difference in exposure to open defecation density between India and SSA is large: sufficiently large that the effect size does not have to be very large to predict a counterfactual change as large as the India-Africa height gap. This is in part because the difference in population density is large, but section 5.1 verifies that population density itself does not explain the gap. Children from India come from richer households, and have substantially fewer siblings, on average. Children in India have better educated-fathers but less-well-educated mothers, a fact consistent with the low social status of women in India.

### 3.2 Nonparametric reweighting method

The non-parametric demographic projection computes a new mean for the Indian sample after reweighting to match the African sample's distribution of a set of observable independent variables. DiNardo et al. (1996) introduced this method to economics in order to decompose difference in wages in labor markets. It has since been applied to demographic contexts, such as to decompose the U.S. racial difference in life expectancy (Geruso, 2012), or to estimate the body mass of immediately pre-pregnancy women in India (Coffey, 2015).

In particular, the approach is to construct a counterfactual mean height of Indian children that matches the distribution of exposure to open defecation among African children:

∙ First, partition both samples into groups *g* ∈ *G*(*X*), which share values or ranges of values of a set of independent variables *X*. In this case, construct 11 categories: 10 deciles of positive local open defecation density, plus an extra category for zero local open defecation (in the child's PSU).
∙ Next, for each group *g*, for each region, compute $f\left(g|s\right)=\frac{\sum_{i\in g}w_{is}}{\sum_{g\in G\left(X\right)}\sum_{i\in g}w_{is}}$, the empirical density within each sample *s* ∈ {India, Africa} in group *g*, using an observation-specific weight *w*_*is*_ for observation *i* in sample *s*. In the main results, each observation receives a weight of 1, following the recommendation of the DHS manual^12^ not to use sampling weights for analysis of relationships; in the supplementary appendix, table A.4 verifies that the main result is qualitatively unchanged if DHS sampling weights are used, instead.
∙ Finally, compute the counterfactual mean height of Indian children
  (2) $${\tilde{h}}_{\text{India}}=\underset{g\in G\left(X\right)}{\sum }\underset{i\in g}{\sum }\frac{f\left(g|\text{Africa}\right)}{f\left(g|\text{India}\right)}w_{i}h_{i},$$

where *h*_*i*_ is the height-for-age *z*-score of child *i* in the Indian sample.

∙ The projected increase in mean child height is the difference between the reweighted Indian mean and the observed Indian mean.

This approach can be combined with other covariates, playing a role analogous to regression controls, by suitable selection of *X*. In particular, to control for a partitioning of the data by bins of another observed property *U*, repeat this procedure twice:

∙ First, project a counterfactual Indian height reweighting only to match the African distribution of *U*.
∙ Next, project Indian height again, but reweighting on categories that are the intersection of *U* and the 11 open defecation categories.
∙ The projected increase in mean height due to open defecation is the difference between these two reweighted means.

## 4 Main result: open defecation density and the India-Africa gap

**Fig. 2:**
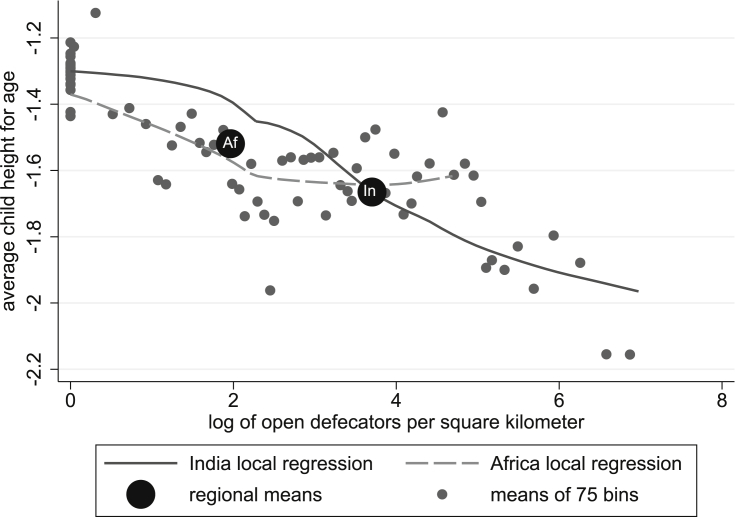
Within-region sanitation gradients can account for India-Africa gap.

How does average height in India and SSA differ, conditional on the same exposure to open defecation density? Fig. 2 plots the data, to permit a visual comparison. The two large dots are the Indian and SSA averages: Indian children are, indeed, shorter on average and exposed to more open defecation. The many small dots plot the data non-parametrically by splitting the sample into 75 equal sample size bins along the horizontal axis of sanitation exposure and computing the averages for each bin: as the downward trend shows, children who are exposed to more and nearer open defecation are shorter on average.

The two curves plot local kernel regressions within the Indian and African samples. Three facts are relevant. First, both similarly slope down. Second, the African line stops: the *maximum* observation of open defecation density in the African sample is at about the 75th percentile of the Indian sample. Third, for much of the common support, and specifically at the point of the average African exposure, the Indian line is on top, indicating that at the African mean level of exposure to open defecation, Indian children are at least as tall as African children, on average.

The rest of this section presents the main result: projections of the increase in the average height-for-age of Indian children that would result from matching the African distribution of density of open defecation, computed for various sub-samples with various additional covariates. First, section 4.1 presents visual summaries of the reweighted result, and then section 4.2 reports the projected counterfactual changes in child height.

### 4.1 Visual summaries

**Fig. 3:**
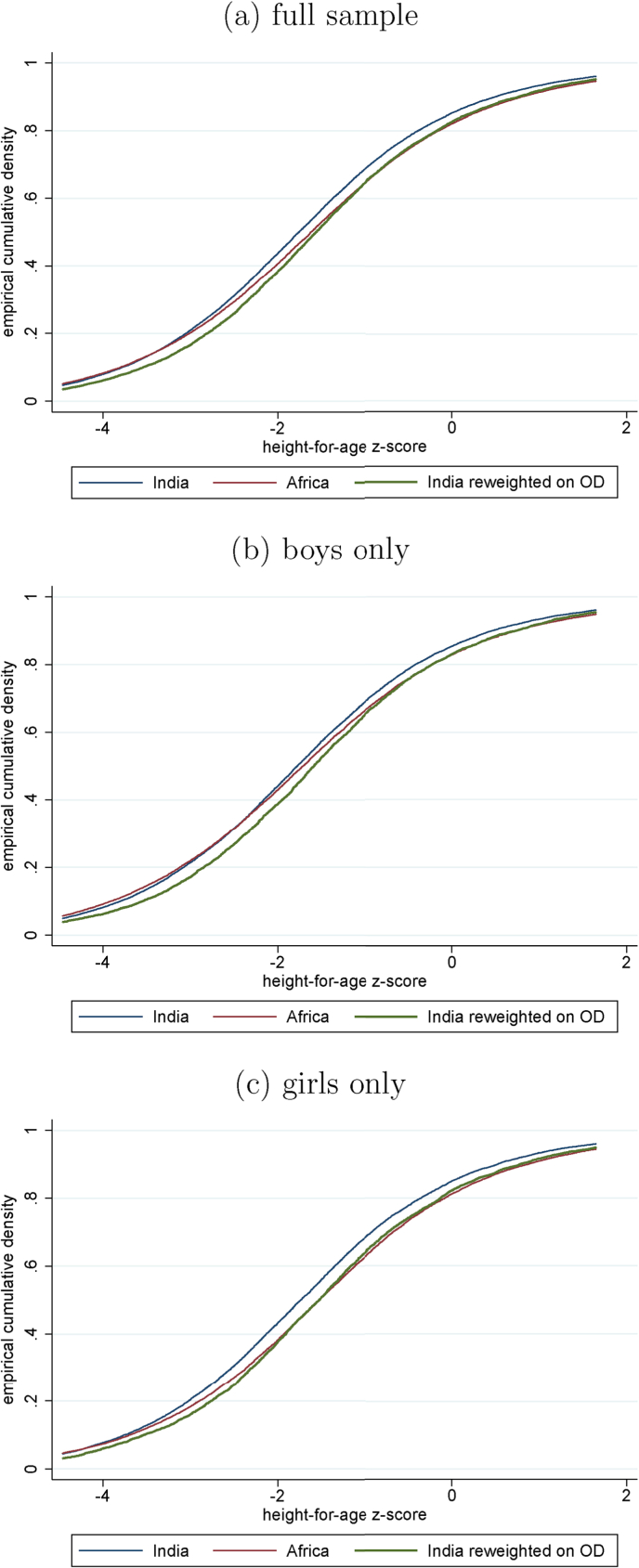
Empirical and reweighted CDFs of child height-for-age, by sex.

An advantage of the DiNardo et al. reweighting method is that, because it matches the full distribution of the target variable and not merely the mean, a full counterfactual distribution can be produced. This is done in Fig. 3. The figure plots the observed CDF of child height in India, the observed CDF of child height in Africa, and a counterfactual Indian CDF reweighted to match the African distribution to exposure to open defecation. In general, the Indian CDF shifts right by approximately as much as the African advantage, and in some cases by more.

**Fig. 4:**
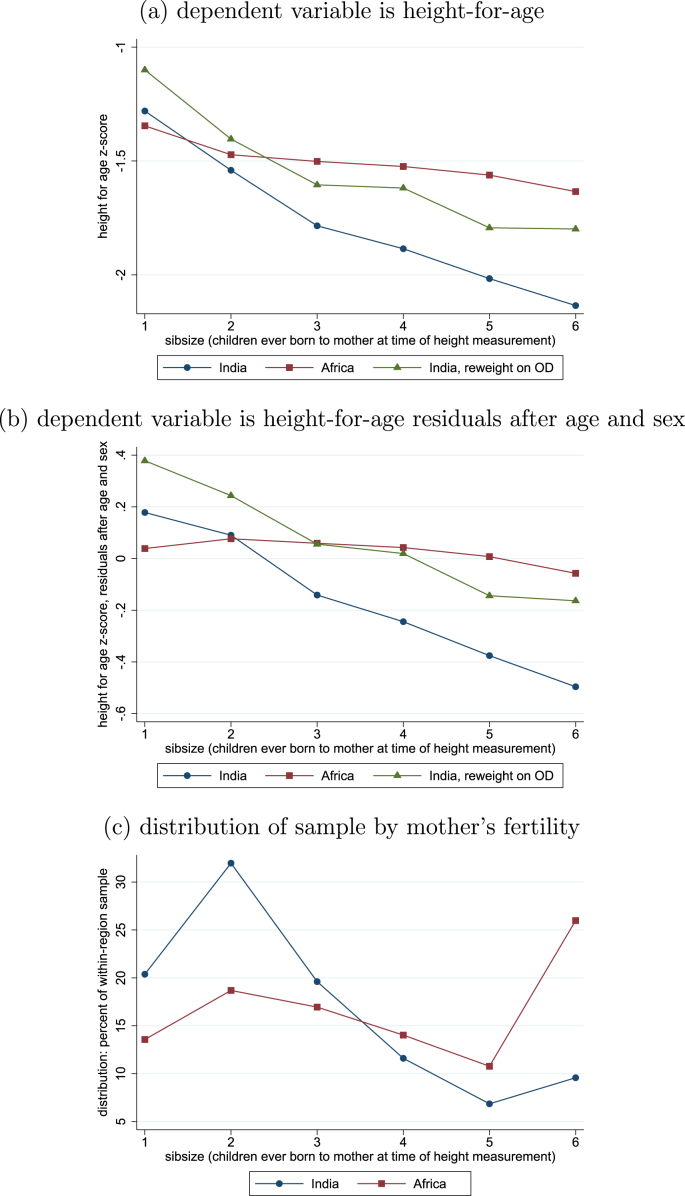
Empirical and reweighted average height-for-age, by mother's fertility.

SSA is at a different point than India in the fertility transition, a stylized fact with wide-ranging consequences for demographic properties of children (Kohler, 2012). If mothers' fertility is correlated with children's height, an analysis which overlooks this fact may misdiagnose the height gap. This is especially important because height-for-age is correlated with child age, and two populations with different fertility and mortality patterns will have a different age distribution among children under five. Fig. 4 presents observed and counterfactually projected average child heights within categories of mothers' fertility, measured as the number of siblings of the child who had been born by the time of the survey. It is clear from panel (c) that fertility is importantly higher in SSA than in India.

Fig. 4 shows that there is a steep negative association between average child height and mother's fertility within India but not in SSA. In a preview of our full results, the reweighting projects an increase in the average height of Indian children of about 0.2 at all levels of fertility, an increase greater than the India-Africa height gap. Because the DHS only measures the height of children under five, birth order and sibsize are highly correlated among the children with measured height.^13^ Section B.2 of the supplementary appendix includes descriptive summary statistics for India and SSA by birth order and sibsize at the time of the survey: children in India whose mothers have had more children have shorter mothers, on average; a slight pattern in the opposite direction is found in SSA. Because of these important correlations in the data, Fig. 4 plots reweighting results by sibsize, and further results below will verify that patterns robustly appear *within* these demographic groups.

### 4.2 Full reweighting results

Table 2, Table 3, Table 4 present our main result: reweighting the sample of Indian children's height to match the distribution of African exposure to open defecation density projects an increase in mean height greater than the India-Africa height gap. Table 2 studies all ages with measured height; Table 3 restricts the sample to ages 6–24 months; and Table 4 restricts the sample to months 25–59, where height-for-age is not correlated with age. Within a table, each row is a separate set of additional covariates according to which the Indian sample is reweighted to match the African sample before incorporating open defecation; thus, these act analogously to regression controls, but they are allowed to non-linearly interact with one another. The numbers presented are the change in projected mean height between reweighting on the controls only and reweighting on open defecation and the controls both.

**Table 2:** Main result: Indian children reweighted to match African sample on open defecation and other properties (all ages).

sex	sibsize at survey	birth order	assets	urban	mom literate	dad any education	mom age at birth	increases in average height from matching African open defecation density ▪ India-Africa z-score gap = 0.146							*p*-value (*n*=7 rank corr. w/ΔOD⋆)
								full sample	first borns	later borns	sibsize of 1 or 2 at time of survey	sibsize > 2 at time of survey	first born boy	other than first born boy	
								0.212	0.177	0.221	0.154	0.247	0.156	0.221	0.007
✓								0.213	0.179	0.221	0.157	0.246	0.156	0.220	0.003
	✓							0.230	0.171	0.244	0.153	0.267	0.154	0.239	0.007
✓	✓							0.228	0.176	0.241	0.154	0.263	0.154	0.237	0.003
✓	✓		✓					0.203	0.171	0.209	0.140	0.231	0.147	0.209	0.016
✓	✓		✓	✓				0.229	0.159	0.247	0.140	0.269	0.153	0.239	0.007
✓	✓		✓	✓	✓			0.193	0.163	0.204	0.125	0.221	0.147	0.199	0.007
✓	✓		✓	✓	✓	✓		0.194	0.201	0.185	0.147	0.206	0.144	0.202	0.072
✓	✓		✓	✓	✓	✓	✓	0.170	0.137	0.178	0.112	0.184	0.127	0.175	0.007
		✓						0.230	0.177	0.243	0.155	0.264	0.156	0.239	0.007
✓		✓						0.230	0.179	0.243	0.156	0.263	0.156	0.239	0.007
✓		✓	✓					0.206	0.161	0.217	0.136	0.233	0.125	0.215	0.003
✓		✓	✓	✓				0.220	0.152	0.238	0.146	0.259	0.133	0.230	0.003
✓		✓	✓	✓	✓			0.184	0.137	0.195	0.127	0.218	0.102	0.193	0.003
✓		✓	✓	✓	✓	✓		0.182	0.210	0.173	0.154	0.200	0.132	0.187	0.230
✓		✓	✓	✓	✓	✓	✓	0.165	0.125	0.172	0.116	0.186	0.096	0.173	0.007
✓	✓	✓	✓	✓	✓	✓	✓	0.184	0.137	0.189	0.134	0.191	0.127	0.189	0.007
			✓					0.203	0.171	0.206	0.147	0.233	0.125	0.210	0.007
			✓	✓				0.209	0.159	0.223	0.138	0.253	0.133	0.214	0.003
			✓	✓	✓			0.166	0.138	0.180	0.119	0.203	0.102	0.171	0.003
			✓	✓	✓	✓		0.158	0.179	0.166	0.149	0.206	0.132	0.165	0.035
			✓	✓	✓	✓	✓	0.155	0.095	0.162	0.086	0.207	0.096	0.163	0.035

*n* (children in India)								42,481	13,445	29,036	22,238	20,243	6,824	35,657	
mean density of OD in India								3.71	3.45	3.87	3.33	4.12	3.34	3.78	
mean density of OD in Africa								1.95	1.81	1.98	1.79	2.02	1.83	1.96	
⋆ difference, India minus Africa								1.76	1.64	1.89	1.54	2.10	1.51	1.82	

*Note:* Columns correspond to sub-samples. Rows correspond to sets of other properties. Differences presented are the difference between the height of Indian children reweighted to match African children on that row's other properties and the height of Indian children reweighted to match the African joint distribution of open defecation and the same other properties. The *p*-value in each row corresponds to the two-sided Kendall's *τ* non-parametric test of a relationship between the 7 results and the 7 differences in open defecation density between India and Africa within each sub-sample, testing the hypothesis that the effect of reweighting is larger in sub-samples where the difference in open defecation density is larger.

**Table 3:** Indian children reweighted to match African sample on open defecation and other properties (ages 6–24 mo).

sex	sibsize at survey	birth order	assets	urban	mom literate	dad any education	mom age at birth	increases in average height from matching African open defecation density ▪ India-Africa z-score gap = 0.131							*p*-value (*n*=7 rank corr. w/ΔOD ⋆)
								full sample	first borns	later borns	sibsize of 1 or 2 at time of survey	sibsize > 2 at time of survey	first born boy	other than first born boy	
								0.260	0.189	0.295	0.202	0.342	0.171	0.278	0.003
✓								0.263	0.185	0.292	0.210	0.339	0.171	0.276	0.003
	✓							0.307	0.190	0.339	0.204	0.374	0.176	0.322	0.003
✓	✓							0.301	0.185	0.332	0.204	0.363	0.176	0.316	0.003
✓	✓		✓					0.269	0.206	0.289	0.212	0.301	0.213	0.277	0.016
✓	✓		✓	✓				0.300	0.228	0.324	0.250	0.323	0.264	0.310	0.035
✓	✓		✓	✓	✓			0.257	0.199	0.274	0.210	0.277	0.233	0.267	0.016
✓	✓		✓	✓	✓	✓		0.273	0.245	0.279	0.235	0.277	0.300	0.274	0.548
✓	✓		✓	✓	✓	✓	✓	0.258	0.175	0.287	0.220	0.267	0.248	0.264	0.035
		✓						0.305	0.189	0.336	0.205	0.371	0.171	0.320	0.003
✓		✓						0.304	0.185	0.335	0.205	0.371	0.171	0.319	0.003
✓		✓	✓					0.291	0.209	0.312	0.222	0.325	0.218	0.300	0.007
✓		✓	✓	✓				0.300	0.229	0.318	0.264	0.314	0.251	0.306	0.016
✓		✓	✓	✓	✓			0.239	0.207	0.246	0.222	0.245	0.232	0.240	0.035
✓		✓	✓	✓	✓	✓		0.266	0.254	0.266	0.253	0.271	0.296	0.262	0.548
✓		✓	✓	✓	✓	✓	✓	0.253	0.162	0.277	0.224	0.265	0.212	0.259	0.016
✓	✓	✓	✓	✓	✓	✓	✓	0.265	0.175	0.287	0.233	0.266	0.248	0.267	0.072
			✓					0.261	0.202	0.283	0.215	0.326	0.218	0.274	0.016
			✓	✓				0.302	0.206	0.336	0.241	0.361	0.251	0.312	0.016
			✓	✓	✓			0.250	0.159	0.282	0.187	0.279	0.232	0.262	0.035
			✓	✓	✓	✓		0.257	0.226	0.272	0.200	0.299	0.296	0.259	0.230
			✓	✓	✓	✓	✓	0.199	0.178	0.216	0.178	0.231	0.212	0.194	0.133

*n* (children in India)								13,487	4,439	9,048	8,249	5,238	2,262	11,225	
mean density of OD in India								3.71	3.39	3.86	3.42	4.16	3.36	3.78	
mean density of OD in Africa								1.93	1.79	1.97	1.80	2.01	1.79	1.95	
⋆ difference, India minus Africa								1.77	1.60	1.89	1.61	2.15	1.57	1.83	

*Note:* Columns correspond to sub-samples. Rows correspond to sets of other properties. Differences presented are the difference between the height of Indian children reweighted to match African children on that row's other properties and the height of Indian children reweighted to match the African joint distribution of open defecation and the same other properties. The *p*-value in each row corresponds to the two-sided Kendall's *τ* non-parametric test of a relationship between the 7 results and the 7 differences in open defecation density between India and Africa within each sub-sample, testing the hypothesis that the effect of reweighting is larger in sub-samples where the difference in open defecation density is larger.

**Table 4:** Indian children reweighted to match African sample on open defecation and other properties (ages 25–59 mo).

sex	sibsize at survey	birth order	assets	urban	mom literate	dad any education	mom age at birth	increases in average height from matching African open defecation density ▪ India-Africa z-score gap = 0.079							*p*-value (*n*=7 rank corr. w/ΔOD ⋆)
								full sample	first borns	later borns	sibsize of 1 or 2 at time of survey	sibsize > 2 at time of survey	first born boy	other than first born boy	
								0.196	0.174	0.193	0.131	0.217	0.133	0.203	0.035
✓								0.196	0.179	0.193	0.135	0.217	0.133	0.203	0.016
	✓							0.202	0.166	0.213	0.128	0.230	0.130	0.212	0.007
✓	✓							0.201	0.176	0.210	0.131	0.227	0.130	0.210	0.007
✓	✓		✓					0.171	0.166	0.169	0.132	0.184	0.124	0.173	0.016
✓	✓		✓	✓				0.179	0.125	0.194	0.081	0.212	0.103	0.187	0.007
✓	✓		✓	✓	✓			0.143	0.146	0.148	0.096	0.156	0.145	0.144	0.133
✓	✓		✓	✓	✓	✓		0.167	0.213	0.153	0.153	0.164	0.137	0.173	0.548
✓	✓		✓	✓	✓	✓	✓	0.150	0.165	0.139	0.122	0.145	0.126	0.154	0.764
		✓						0.195	0.174	0.200	0.136	0.219	0.133	0.203	0.007
✓		✓						0.198	0.179	0.202	0.141	0.220	0.133	0.205	0.007
✓		✓	✓					0.186	0.154	0.194	0.128	0.200	0.113	0.194	0.007
✓		✓	✓	✓				0.182	0.122	0.196	0.107	0.218	0.091	0.192	0.003
✓		✓	✓	✓	✓			0.132	0.104	0.138	0.100	0.155	0.073	0.138	0.007
✓		✓	✓	✓	✓	✓		0.161	0.214	0.145	0.179	0.156	0.100	0.167	0.764
✓		✓	✓	✓	✓	✓	✓	0.147	0.138	0.144	0.129	0.138	0.091	0.152	0.230
✓	✓	✓	✓	✓	✓	✓	✓	0.156	0.165	0.144	0.161	0.136	0.126	0.156	0.368
			✓					0.180	0.166	0.176	0.131	0.195	0.113	0.186	0.016
			✓	✓				0.162	0.129	0.174	0.085	0.203	0.091	0.172	0.007
			✓	✓	✓			0.127	0.128	0.144	0.079	0.154	0.073	0.135	0.007
			✓	✓	✓	✓		0.122	0.197	0.130	0.128	0.151	0.100	0.136	0.230
			✓	✓	✓	✓	✓	0.127	0.112	0.127	0.074	0.153	0.091	0.141	0.035

*n* (children in India)								25,329	7,833	17,496	11,802	13,527	3,994	21,335	
mean density of OD in India								3.68	3.29	3.86	3.21	4.09	3.29	3.76	
mean density of OD in Africa								1.95	1.82	1.99	1.77	2.02	1.85	1.96	
⋆ difference, India minus Africa								1.73	1.48	1.87	1.44	2.07	1.44	1.79	

*Note:* Columns correspond to sub-samples. Rows correspond to sets of other properties. Differences presented are the difference between the height of Indian children reweighted to match African children on that row's other properties and the height of Indian children reweighted to match the African joint distribution of open defecation and the same other properties. The *p*-value in each row corresponds to the two-sided Kendall's *τ* non-parametric test of a relationship between the 7 results and the 7 differences in open defecation density between India and Africa within each sub-sample, testing the hypothesis that the effect of reweighting is larger in sub-samples where the difference in open defecation density is larger.

Each column is a subsample of children, with splits chosen with attention to demographic categories that are potentially relevant for child height in India: birth order, mother's fertility, and the interaction of sex and birth order. Because children in the subsamples live in different contexts, the three rows along the bottom show that the India-SSA difference in exposure to open defecation is different across the seven subsamples. The column of *p*-values along the far right-hand side of the table presents separate statistical significance tests for each row. These are each a *p*-value on a non-parametric two-sided Kendall's *τ* test with *n* = 7, testing that across columns the projected difference in mean height is associated with the size of the gap in open defecation for that subsample. In Table 2's full sample, in all rows but one the test results indicate clear statistical significance.

The number of children that a child's mother has had by the time of the DHS survey is an important predictor of household socioeconomic status in India. For example, measured second-born Indian children with one other sibling have a mother who is 152.3 cm tall, on average, compared with 151.7 cm among second-born Indian children with two or more siblings when their height is measured. Therefore, in addition to the reweighting controls, the robustness of the result to restricting the sample along these important dimensions is a meaningful indicator that the apparent importance of open defecation externalities does not simply reflect confounding. Taken together, the 462 separately-computed projections collectively and individually suggest that the increase in the height of Indian children would be at least as great as the India-Africa height gap, if Indian children were exposed to the African distribution of open defecation.

In the supplementary appendix, table A.5 presents reweighting results separately for girls and boys. Although the projected counterfactual increase is a little greater for girls than for boys, results are comparable across sexes, and in both cases the projected increase is about as large or larger than the overall India-SSA gap. One interesting note is that the India-SSA gap is larger for girls than for boys. This coheres with the observation of Barcellos et al. (2014) that the fact that girls have about the same average height-for-age as boys in India is a marker of relative disadvantage, because height-for-age tends to be less negative among girls than boys within other developing countries.^14^ In our case, table A.5 is consistent with a general observation of this paper, presented in section 4.1 with a focus on sibsize: although mean height-for-age predictably differs across demographic groups within India (which is a population where social and demographic forces are important determinants of health outcomes) there is a robust pattern in which open defecation is similarly quantitatively important within these groups.

## 5 Extensions

### 5.1 Robustness check: linear regression results

The decomposition results in section 4 are limited by a curse of dimensionality: with too many covariates, observations must be dropped to perform the reweighting, because there are no Indian observations that match a category in the SSA sample. This is a particular challenge given the need to control for age, or to split the sample into age categories. This section presents robustness checks using regression, assuming a constant linear association between open defecation density and child height, but allowing more controls. Thus, this section estimates:

(3) $$height_{ips}=\alpha India_{s}+\beta \ln\;{\left(ope\;defecation\;density+1\right)}_{ps}+X_{ips}\theta +R_{ips}+{ɛ}_{ips},$$

where *height*_*ips*_ is the height-for-age *z*-score of child *i* in survey primary sampling unit (PSU) *p* in sample *s*, which is either India or SSA, with or without 288 sub-national region fixed effects *R*_*ips*_ and a vector of controls *X*, including for age-in-months. *India*_*s*_ is an indicator that the child is from India, rather than SSA. Standard errors are clustered by PSU. The coefficient of interest is *β* on exposure to local open defecation. This variable, in contrast to a simple indicator for a child's household's own open defecation, has the advantage of reflecting sanitation externalities.

Table 5 presents estimates of *α* and *β* from regression equation (3), including age-restricted sub-samples in panels B and C. The top row presents $\hat{\alpha }$, the India-Africa height gap, after linearly accounting for open defecation density; it is missing in columns 4 and 9, where fixed effects for sub-national regions are used. It is never negative once open defecation density is accounted for. However, $\hat{\alpha }$ is essentially unchanged in column 7 when population density (rather than open defecation density) is controlled for, suggesting that this result is not due to population density itself.

**Table 5:** Open defecation density accounts for the height gap: OLS regression as decomposition.

sample:	(1)	(2)	(3)	(4)	(5)	(6)	(7)	(8)	(9)
	full	full	full	full	1st boys	not 1st boys	full	full	full
Panel A: All ages 0–59 months									
India	−0.146∗∗∗ (0.0156)	0.0167 (0.0163)	0.0808∗∗∗ (0.0162)		0.318∗∗∗ (0.0337)	0.104∗∗∗ (0.0188)	−0.136∗∗∗ (0.0163)	0.0835∗∗∗ (0.0167)	
ln(OD density)		−0.0922∗∗∗ (0.00376)	−0.0946∗∗∗ (0.00375)	−0.0988∗∗∗ (0.00438)	−0.0587∗∗∗ (0.00680)	−0.0468∗∗∗ (0.00383)		−0.0945∗∗∗ (0.00376)	−0.0523∗∗∗ (0.00424)
density							−0.0000193∗ (0.00000849)	−0.00000581 (0.00000690)	
*n* (children)	170,149	170,149	170,149	170,149	19,232	146,698	170,149	170,149	165,932
projected Δh.f.a.		0.160	0.164	0.172	0.102	0.091		0.157	0.100
Panel B: Ages 6–24 months									
India	−0.131∗∗∗ (0.0220)	0.0329 (0.0234)	0.0666∗∗ (0.0228)		0.302∗∗∗ (0.0573)	0.116∗∗∗ (0.0280)	−0.107∗∗∗ (0.0233)	0.0830∗∗∗ (0.0237)	
ln(OD density)		−0.0924∗∗∗ (0.00539)	−0.0943∗∗∗ (0.00529)	−0.0893∗∗∗ (0.00627)	−0.0625∗∗∗ (0.0117)	−0.0525∗∗∗ (0.00568)		−0.0936∗∗∗ (0.00529)	−0.0460∗∗∗ (0.00638)
density							−0.0000502∗∗∗ (0.0000150)	−0.0000362∗∗ (0.0000136)	
*n* (children)	57,494	57,494	57,494	57,494	6658	49,753	57,494	57,494	56,411
projected Δh.f.a.		0.161	0.164	0.156	0.110	0.102		0.155	0.088
Panel C: Ages 25–59 months									
India	−0.0786∗∗∗ (0.0175)	0.0924∗∗∗ (0.0183)	0.0966∗∗∗ (0.0183)		0.312∗∗∗ (0.0414)	0.0774∗∗∗ (0.0212)	−0.0726∗∗∗ (0.0182)	0.0957∗∗∗ (0.0188)	
ln(OD density)		−0.0988∗∗∗ (0.00424)	−0.0990∗∗∗ (0.00426)	−0.115∗∗∗ (0.00506)	−0.0607∗∗∗ (0.00825)	−0.0391∗∗∗ (0.00433)		−0.0990∗∗∗ (0.00426)	−0.0626∗∗∗ (0.00492)
density							−0.0000116 (0.00000926)	0.00000184 (0.00000800)	
*n* (children)	94,906	94,906	94,906	94,906	10,570	81,479	94,906	94,906	92,051
projected Δh.f.a.		0.169	0.169	0.197	0.103	0.075		0.162	0.118
age × sex FEs			*✓*	*✓*	*✓*	*✓*	*✓*	*✓*	*✓*
region FEs				*✓*					*✓*
extended controls					*✓*	*✓*			*✓*

*Note:* Dependent variable: height-for-age *z*-score. Standard errors clustered by PSU: †*p* < 0.10; ∗*p* < 0.05; ∗∗*p* < 0.01; ∗∗∗*p* < 0.001. 288 region fixed effects are for DHS sub-national regions (v024). Extended controls are mother's height and indicator sets for household asset count, sibsize at the survey, birth order, multiple births, month of birth, mother's literacy, father's education, and whether the child was immediately breastfed.

Columns 5, 6, and 9 include a further set of extended controls: mother's height in centimeters and sets of indicators for household asset count, sibsize at the time of the survey, birth order, whether the child was a multiple birth, month of birth, mother's literacy, father's education, and whether the child was immediately breastfed. For each sub-sample and set of controls, the bottom three rows compute the linearly-projected counterfactual increase in average child height from moving to the mean of open defecation density in the SSA sample.^15^ Each case suggests that the difference in exposure to open defecation density projects a large difference in child height-for-age. In particular, in columns with controls for age fixed effects, the projected difference should be compared with the smaller Asian enigma once the difference in ages between the Indian and SSA samples is controlled for, which is about 0.09 in the all-ages sample (see footnote 3).

### 5.2 Mechanism: could there be an effect through maternal nutrition?

Section 2.2 introduced two mechanisms through which exposure to open defecation could impact child height: through the disease and net nutrition experienced by the child, or through the health of the mother, during and before pregnancy (Institute of Medicine, 1990; Behrman et al., 2009; Prendergast et al., 2014; Padhi et al., 2015; Duh and Spears, 2017). This section presents suggestive evidence in support of the second possibility by showing that open defecation density predicts the body mass of the mothers of the children in our height sample. An effect through mothers would be consistent with the early-age height differences seen in Fig. 1, as well as with the effects of open defecation on neonatal mortality documented by Geruso and Spears (2018). As Coffey and Spears (2018b) detail, the fact that mothers in our data are weighed at the time of the DHS survey, rather than in pregnancy, limits the clarity with which conclusions can be drawn about effects on children *in utero* and during breastfeeding. The observation here is only that the body mass of mothers is predicted by exposure to open defecation's infectious disease.

Table 6 presents regressions of mothers' body mass index (BMI) on the open defecation density dependent variable and other controls, in regressions where observations are the same children as in the height sample. As columns 1 and 2 document, Indian mothers are substantially more likely to be underweight than mothers in SSA. Much of this difference is due to nutritional consequences of women's social status in India (Das Gupta, 1995; Coffey et al. 2017a,b; 2018), but column 2 suggests that some minority fraction of the difference is due to the disease environment, as also pointed to by (Coffey, 2015) observation that even a quarter of working-age adult men are underweight in India. The rightmost column confirms that a similarly-sized coefficient on open defecation persists, even with controls for other well-studied predictors of adult weight in India.

**Table 6:** Open defecation density predicts the body mass of mothers.

	(1)	(2)	(3)	(4)	(5)
	Dependent variable: Mother's BMI				
India	−2.057∗∗∗ (0.0402)	−1.437∗∗∗ (0.0412)			
ln(OD density)		−0.351∗∗∗ (0.00998)	−0.436∗∗∗ (0.0114)	−0.292∗∗∗ (0.0109)	−0.262∗∗∗ (0.0108)
mother's height					−0.0600∗∗∗ (0.00313)
months since last birth					−0.00545∗∗∗ (0.00101)
currently pregnant					0.791∗∗∗ (0.0355)
currently breastfeeding					−0.435∗∗∗ (0.0336)
sub-national region FEs			*✓*	*✓*	*✓*
asset indicators				*✓*	*✓*
extended BMI controls					*✓*
constant	22.42∗∗∗ (0.0232)	23.10∗∗∗ (0.0306)			
*n* (child-mothers)	168,632	168,632	168,632	168,632	168,628

*Note:* Observations are children in the main height sample, even though the dependent variable is the Body Mass Index (BMI) of their mother. Standard errors are clustered by survey PSU in parentheses. †*p* < 0.10; ∗*p* < 0.05; ∗∗*p* < 0.01; ∗∗∗*p* < 0.001. Region fixed effects are 288 fixed effects for DHS sub-national regions (v024). Asset indicators are fixed effects for the count (0–5) of assets, summarized in Table 1. The extended BMI controls are fixed effects indictors, each interacted with an indicator for India, for: sibsize at the time of the survey (6 × 2 categories), the mother's age in years at the time of the measurement (35 × 2 categories), and whether the mother is literate (2 × 2 categories). For discussion of these predictors of maternal BMI in India and SSA, see Coffey (2015).

An important caveat is that if what matters is the exposure of a mother to open defecation externalities in her childhood, rather than during pregnancy, then such exposure is not well-measured by our independent variable. Open defecation, we have emphasized, is changing only slowly in India (Coffey and Spears, 2018c), but it has changed over decades, and most Indian women marry into villages and neighborhoods related to, but not identical to, those where they grew up. BMI, however, is a measure of *recent* or short-term net nutrition, such as could be influenced by diarrhea or enteropathy. Moreover, column 4 controls for the mother's height, which would reflect her own early-life environment, and still finds an association between open defecation and BMI.

## 6 Meta-analysis: evidence from recent studies in the literature

Would counterfactually causing Indian children to be exposed to the African distribution of open defecation increase their height by an amount as large as the India-Africa height gap? To settle this question would require an estimate of what the average effect of open defecation on child height is in India. In addition to the ordinary difficulties of estimating causal effects, such an estimate would have to take into consideration two special factors: first, *externalities*, because children in India are harmed by their neighbors' open defecation in addition to their own household's (Geruso and Spears, 2018); and second, *parameter heterogeneity*, because the average effect of open defecation within India may be different from the average effect in other places, for example because of high population density even in rural India, which increases epidemiological externalities.

Although no such estimate exists that is plausibly representative of India and causally well-identified, since the original circulation of the working paper version of this paper, a number of related estimates have emerged of the effect of local open defecation externalities on child height, in a variety of contexts. This section reviews and integrates estimates of the effect of the fraction of a child's PSU neighbors who defecate in the open on her height-for-age, measured in standard deviations of a reference population. Section A in the supplementary appendix further considers related recent estimates of other parameters, in papers that do not specifically estimate an effect of sanitation externalities on child height-for-age.

Table 7 summarizes studies that report an estimate of the effect of *the fraction of a child's neighbors who defecate in the open* on her *height-for-age*, or report an association between these two variables, as well as two related cross-sectional studies. These differ from the ideal evidence in at least two ways. First, many of the studies are not from India; the effect of open defecation in these contexts may differ. Second, none of them consider the role of population density. Nevertheless, they are informative.

**Table 7:** Estimates of the effect of open defecation on child height from the literature, and implications for the height gap.

	context	identification	coefficient	95% CI	% DHS gap	% JMP gap
**randomized experiments**						
Gertler et al. (2015)	India, Indonesia, Mali	IV from three RCTs	−0.46	(-0.772, −0.148)	98	85
Clasen et al. (2014)^a^	India	IV from RCT, see note	0.351	(-0.460, 1.163)	⋅	⋅
**difference in trends**						
Headey (2015)	Ethiopia	region and time FEs	−0.31	(-0.53, −0.09)	66	57
Vyas et al. (2016)	Cambodia	province and time FEs	−0.502	(-0.684, −0.320)	107	93
Spears (2013)	India	district and time FEs	−0.553	(-0.798, −0.308)	118	102
**cross-sectional**						
Jayachandran and Pande (2013)	India and Africa	cross-section, OD	−0.358	(-0.382, −0.334)	76	66
Lin et al. (2013)	Bangladesh	cross-section, WASH	−0.54	(-1.01, −0.06)	115	100
**meta-estimates**						
all seven studies			−0.360	(-0.384, −0.337)	77	67
excluding Jayachandran and Pande (2013)			−0.421	(-0.550, −0.292)	90	78
excluding both cross-sectional and Clasen et al. based IV			−0.436	(-0.572, −0.299)	92	80

*Note:* Coefficients are, unless otherwise noted, coefficients on the local area fraction of households defecating in the open, predicting child height-for-age. The Lin et al. (2013) estimate compares households with dichotomized extreme sanitation environments, in data extracted from an experimental project. The Spears (2013) estimate is taken from Table 6 of section 3 of the World Bank Policy Research Working Paper version of this paper. The Jayachandran and Pande (J & P) (2013) estimate compares households with and without latrines in the child level dataset used in section 4 and is further discussed in supplementary appendix section B.1; it is excluded from some meta estimates because its very small standard errors dominate the averaging. The meta estimate weights estimates by the square of the inverse of their standard error. To explain the India-Africa height gap, the coefficient on local open defecation must be 0.54 using open defecation figures from the Unicef-WHO Joint Monitoring Programme or 0.47 using the same DHS data compilation used in section 4. None of these estimates consider the interaction of open defecation with population density.

a The Clasen et al. (2014) RCT in Orissa, India did not find a large first stage effect on open defecation, and therefore did not find a statistically significant effect on child height. Quoted here with permission are preliminary results from an in-progress reanalysis of the data by Scovronick et al. to produce an IV estimate and confidence interval of the implied effect of local open defecation on child height; the confidence interval is large.

Gertler et al. (2015) report an instrumental variables estimate obtained by instrumenting village average open defecation with treatment status from a randomized impact evaluation of a sanitation program, pooling data from three field experiments: Cameron et al. (2013) in Indonesia, Patil et al. (2014) in Madhya Pradesh, India, and Pickering et al. (2015) in Mali. Their estimate captures local externalities because all three experiments were randomized at the village level and is identified only off of randomized treatment assignment. The point estimate of −0.46 closely matches the required effect size of −0.47 that was computed in the background section using equation (1).^16^ Moreover, it is plausible that the average effect in India is even larger than what they estimate, due to population density. Therefore, the evidence from these three randomized experiments is consistent with open defecation being able to statistically account for much or all of the India-Africa height gap.

Clasen et al. (2014) conducted a randomized experiment in rural Orissa, designed to measure the effect of open defecation on child height.^17^ They did not find an effect on height, which they attribute to the difficultly of generating a large first stage effect on open defecation in rural India.^18^ We quote with permission preliminary results by Scovronick et al., in collaboration with the original researchers, to estimate a confidence interval for the implied IV estimate of the effect of local open defecation on child height. Because the first stage estimate is small, the confidence interval is very large, spanning from very harmful effects, through zero, to include effects large enough to account for the Asian enigma at the −0.46 end of the 95% confidence interval.

The next three estimates study difference-in-differences-type consequences of differential changes in open defecation within countries, using fixed effects for sub-national geographic areas and for change over time. The coefficients are ordered by increasing population density of the country studied at the time studied: Ethiopia's population density in 2000 was 85 percent as large as Cambodia's in 2005 and 22 percent as large as India's in 1992. The 95% confidence interval for each estimate excludes zero and includes an effect size large enough to account for the India-Africa gap.

Two further estimates from the literature reflect cross-sectional, household-level comparisons that are not intended by their authors as causal effects. Lin et al. (2013) compare children living in households in rural Bangladesh with extremely good or bad sanitation and hygiene environments. We include their estimate that includes DHS-like socioeconomic status controls. Their data also allow them to show that exposure to poor sanitation and child height are associated with biologically measured markers of environmental enteric disfunction. Jayachandran and Pande (2013) include an estimate of the association between a household-level open defecation indicator and child height in their pooled India-Africa sample.^19^

The final rows of Table 7 pool these estimates to compute meta-estimates of the effect of local open defecation on child height-for-age. These estimates are formed using the weighted least squares approach described by Hedges and Olkin (1985) and recently by Becker and Wu (2007) and used, for example, by Bini et al. (2001), in which each estimate is weighted by the inverse of the variance of its sampling error. By assumption, this procedure ignores the possibility of parameter heterogeneity. Insofar as such heterogeneity is important and due to population density, this procedure would produce an underestimate of the ability of open defecation to explain the height gap.

The first pooled estimate very closely matches the point estimate and confidence interval of the Jayachandran and Pande (2013) estimate, because its sample size is much larger than the other studies', so it dominates the weighted average. However, this estimate was not intended as a causal effect, does not consider externalities from other households, and is largely estimated from African children who are exposed to low population density. The final two rows contain meta-estimates from the non-cross-sectional studies. These are all quantitatively close to the Gertler et al. (2015) IV estimate, and to the magnitude necessary to explain the India-Africa height gap; if population density indeed interacts with open defecation, that would be a reason to consider this an underestimate for India. The reasoning in this section illustrates one valuable complementarity between experimental and demographic research (Gertler et al., 2015): experimental estimates are given more usefulness and import by situating their quantitative magnitude within the context of data on height and sanitation that is representative of the populations of interest.

## 7 Conclusion

This paper responds to a puzzle in the literature on early-life human capital, one that has attracted much attention from economists: why are children in India shorter than children in sub-Saharan Africa? This study presented evidence from a set of demographic projections of mean height in India, under African levels of sanitation. Open defecation can statistically account for much or all of this height difference, in the sense that children in India are projected to be at least around 0.14 standard deviations taller if counterfactually exposed to the African distribution of open defecation density.

However, the age-adjusted India-Africa gap is even smaller than this — and, with or without age adjusting, the gap is a small fraction (no more than 10%) of India's overall height deficit relative to a healthy population. So, one understanding of this paper is that the difference in exposure to open defecation between India and Africa is so large that the size of open defecation's effect does not have to be very large to be able to account for the difference. This paper has not made a precise claim about the exact magnitude of the effect size, in part because it is almost certainly different in different contexts. Still, especially in light of the high population density in even rural India, estimates in the literature and in this paper suggest that the effect of open defecation on child height in India is unlikely to be small: we conclude that exposure to open defecation is among the factors shaping the distribution of child height in India, and that quantitatively its importance is of about the same magnitude as the India-Africa height gap.

Questions remain about the health effects of open defecation: the DHS data do not measure biological pathways such as worm loads or enteric dysfunction, nor the contamination of the environment, food, or water by pathogens from human or animal feces; mothers' weights are measured at the time of the survey, not during pregnancy or breastfeeding, and we do not observe mothers' childhood environments. Therefore, these data cannot distinguish among three hypotheses about the mechanism about open defecation's effect: (1) through the health of children as they are exposed to their environment through the classic “F-diagram” pathways of feet, fingers, flies, fluids, and feet; or through the health of their mothers — as suggested by differences between India and SSA at young ages — which could occur through either (2) near-term body-mass-type mechanisms (for which section 5.2 provides suggestive evidence) or (3) longer-term height-type mechanisms that would be difficult to influence in a short-term intervention study. There is no reason to believe that these mechanisms could not be at work simultaneously; none of these possibilities is ruled out. Finally, the facts of this paper of course do not imply that open defecation is the only factor responsible for children in India being shorter than would be healthy: even at African levels of average child height, there would still be well over a height-for-age standard deviation of height shortfall left to explain. Intrahousehold inequality, the low social status of young women, and maternal nutrition are all likely to be implicated in this larger deficit (Coffey and Hathi, 2016).

## Footnotes

1 The DHS does not measure person-level open defecation, but there is little doubt that most Indians defecated in the open during the time period studied in this paper (Coffey and Spears, 2017).

2 Open defecation is now almost eliminated from Bangladesh, a predominately Muslim country which does not share the social determinants of open defecation discussed by Coffey and Spears (2017). At the same level of exposure to open defecation, children in Bangladesh are not taller than equally-rich children in West Bengal, although Bangladeshi advantage can also be statistically accounted for by controlling for mothers' literacy.

3 Although not, to our knowledge, previously noted in the literature, this simple subtraction may be an overestimate of the magnitude of the Asian enigma. This is because average height-for-age decreases in age in developing countries where stunting is common (Victora et al., 2010), and mortality is higher in SSA than in India, so the average child who survives to have height measured is older in India than in SSA in these data – see Fig. 1. However, this consideration suggests that the true effect size needed to account for the height gap may be smaller, because the gap is smaller; with age controls, the gap is −0.09, although this paper often uses the larger gap without age controls where doing so is appropriate and conservative.

4 Hathi et al. (2017) use DHS data from all available countries. For confirmation of the interaction between population density and open defecation in this paper's sample, see supplementary appendix section B.1.

5 On environmental enteric disfunction, see also Lin et al. (2013); Kosek et al. (2013).

6 It is unfortunate but unsurprising that there is little direct empirical evidence in the literature for the hypothesis that a poor disease environment influences maternal net nutrition, which in turn influences intra-uterine growth and child anthropometry. Section 6 discusses difficulties in finding an effect of sanitation environments on children; the intergenerational timing involved in such a study would be even more challenging.

7 According the JMP data for 2015 — which, as Coffey and Spears (2017) discuss, very likely underestimates open defecation in India particularly — eleven countries have a larger fraction defecating in the open than India: Eritrea, South Sudan, Niger, Chad, Burkina Faso, Sao Tome and Principe, the Solomon Islands, Benin, Togo, Namibia, Liberia, and Cambodia. These have a total population of 111 million people, which is 8 percent of India's 1.3 billion.

8 To many general-caste Hindus, emptying their family's latrine pit would be unthinkable; to may low-caste Dalits, increasingly hoping for social equality, the stigma is no longer worth the financial payment offered for doing this degrading work (Coffey et al., 2017a, Coffey et al., 2017b; 2018). In equilibrium, nobody expects a latrine pit to be able to be emptied, so few households are willing to accumulate feces in them, and the obvious widespread practice of open defecation offers social proof both that open defecation is normal and that emptying latrine pits really must be essentially impossible.

9 The surveys are Cameroon 2004, Chad 2004, Congo (Brazaville) 2005, DRC 2007, Ethiopia 2005, Ghana 2008, Guinea 2005, Kenya 2005, Lesotho 2004 and 2009, Liberia 2007, Madagascar 2003, Malawi 2006, Mali 2006, Namibia 2006, Niger 2006, Nigeria 2008, Rwanda 2005, Sao Tome 2008, Senegal 2005, Sierra Leone 2008, Swaziland 2006, Tanzania 2004 and 2010, Uganda 2006, Zambia 2007, Zimbabwe 2005 and 2006. Spears (2013), in an earlier working paper version, finds similar results with a slightly different sample. Section B.3 of the supplementary appendix confirms that the main result of this section is robust to Monte Carlo randomly generated alternative SSA comparison samples that draw random sub-samples of the African DHS surveys.

10 In the decomposition reweighting results, the fact that the log of this explanatory variable is taken does not matter: the reweighting is done over quantile bins, with no assumption of functional forms, or even of a monotonic relationship between open defecation exposure and height. In regression equation (3) the log is used because it fits the data, presumably because of the very skewed distribution of population density. A Box-Cox transformation recommends a log, with the likelihood maximized at *λ* = −0.06 and with *λ* = 1 and *λ* = −1 both rejected by the data.

11 Some households will have moved, but such permanent migration is less common in India than in other developing countries. The 2012 India Human Development Survey asks how long a household has lived where it currently lives: less than 1% of all households (and about 1.1% of households with a child) report having lived where they currently live for less than five years.

13 Jayachandran and Pande (2017), using the same DHS data as this paper, find that higher-birth-order children in India are shorter than lower-birth-order children in India, on average, and that this gradient between birth order and average height is more steeply negative in India than in sub-Saharan Africa. In principle, the results in that paper and this paper do not conflict: because Indian children are much shorter than would be healthy, important birth order patterns and effects of sanitation could coexist. However, Jayachandran and Pande (2017) further interpret their result as a causal effect of birth order. Investigations into child height and family structure in India, described in a working paper by Spears et al. (2018), suggest that this result is sensitive to specification choice: it reverses when one controls for sibsize at the time of the survey. Sibsize at the time of survey is mechanically correlated with birth order. This correlation is especially high in DHS height data because the DHS only observes height for the youngest children. Further, larger sibsize (or higher mother's fertility) is a marker of disadvantage in India, but not in sub-Saharan Africa. Comparisons across birth orders at the same sibsize are standard in the birth order literature (Blake, 1989); however, empirical strategies that are successful elsewhere in the birth order literature (such as mother fixed effects) are inappropriate for use with the DHS height data because of age restrictions in the DHS data (for details, see Spears et al., 2018). Recall from Table 1 that about two-thirds of the DHS height sample is last-born to their mothers at the time of the survey. 97% of those with measured height are the most-recently or second-most-recently born; the comparable figure is 50% in the full birth history (births with or without measured height).

14 A complication is that girls in India with measured height are about 0.42 months younger on average than boys in India with measured height (clustered s.e. = 0.17), which would make them appear taller in *z*-scores, but there is no such difference in SSA (0.10, s.e. = 0.10). This reflects girls' relatively higher mortality rates in India, but over both sexes, the average child in India is about 2 months older (s.e. = 0.085) than the average child in SSA.

15 This is computed as $\hat{\beta }\times \Delta \ln\;{\left(\text{OD}\;\text{density}\right)}^{\text{India}-\text{SSA}}$, where the difference in exposure to open defecation between India and SSA is computed, for each subsample, in a regression of this variable on an indicator for India and the same control variables as used in that column (except for region fixed effects, which would absorb the India indicator).

16 Recall that the denominator for this figure was computed with DHS data. An alternative denominator can be taken from the Unicef-WHO Joint Monitoring Programme (JMP) statistics on open defecation, which report a difference in open defecation of 26.9 percentage points between India and Africa in the year of India's DHS survey. This smaller denominator produces a slightly larger required effect size of 0.54. The smaller difference between India and Africa in the JMP data may be because their methodology averages over estimates of latrine use (such as in the DHS) and latrine ownership (such as in the census). Because it is particularly common in India to own a latrine but nevertheless defecate in the open (Coffey et al., 2014), the JMP methodology will underestimate open defecation in India and therefore underestimate the India-Africa difference. However, we include this estimate as a conservative robustness check of the required effect size.

17 Other recent field experiments on sanitation in Kenya and Bangladesh have investigated the consequences for child height of upgrading lower-quality latrines to higher-quality latrines (Arnold et al., 2013). In both settings, open defecation, which we study here, was very low at baseline. Coffey and Spears (2018a) use DHS data to show that these experiments closely match what is found by running an analogous regression in observational data.

18 The authors conclude: “Insufficient coverage and use of latrines seem to be the most likely causes for the absence of effect, because no evidence showed that the intervention reduced faecal exposure. Although mean coverage of latrines increased substantially in the intervention villages, more than a third of village households (on average) remained without a latrine after the intervention. About twice that many had no functional latrine that was used at the midpoint of the surveillance period … our results show that the health benefits generally associated with sanitation cannot be assumed simply by construction of latrines.”

19 Jayachandran and Pande (2013) use an indicator for household open defecation, rather than the fraction of a child's local neighbors who defecate in the open. Their estimate also pools data from India, where the effect may be large, and Africa, where the effect may be smaller. Section B.3 of the supplementary appendix, using the same dataset, verifies that the gradient between open defecation and child height is steeper in India than in Africa and that local open defecation interacts with population density. The published version of this analysis (Jayachandran and Pande, 2017) instead presents an interaction between birth order and the household-level open defecation indicator.

## Footnotes

12 “Use of sample weights is inappropriate for estimating relationships, such as regression and correlation coefficients” (Rutstein and Rojas, 2006); see also Solon et al. (2013).

## References

[bib1] Alderman Harold, Linnemayr Sebastian (2009). Anemia in low-income countries is unlikely to be addressed by economic development without additional programs. Food Nutr. Bull..

[bib2] Alsan Marcella, Goldin Claudia (2015). Watersheds in Infant Mortality: the Role of Effective Water and Sewerage Infrastructure, 1880 to 1915.

[bib3] Arnold Benjamin F., Null Clair, Luby Stephen P., Unicomb Leanne, Stewart Christine P., Dewey Kathryn G., Ahmed Tahmeed, Ashraf Sania, Christensen Garret, Clasen Thomas (2013). Cluster-randomised controlled trials of individual and combined water, sanitation, hygiene and nutritional interventions in rural Bangladesh and Kenya: the WASH Benefits study design and rationale. BMJ Open.

[bib4] Baird Sarah, Hicks Joan Hamory, Kremer Michael, Miguel Edward (2011). Worms at Work: Long-run Impacts of Child Health Gains.

[bib5] Barcellos Silvia Helena, Carvalho Leandro S., Lleras-Muney Adriana (2014). Child gender and parental investments in India: are boys and girls treated differently?. Am. Econ. J. Appl. Econ..

[bib6] Becker Betsy Jane, Wu Meng-Jia (2007). The synthesis of regression slopes in meta-analysis. Stat. Sci..

[bib7] Behrman Jere R., Calderon Maria C., Preston Samuel H., Hoddinott John, Martorell Reynaldo, Aryeh D Stein (2009). Nutritional supplementation in girls influences the growth of their children: prospective study in Guatemala. Am. J. Clin. Nutr..

[bib8] Bhalotra Sonia, Valente Christine, Arthur Van Soest (2010). The puzzle of Muslim advantage in child survival in India. J. Health Econ..

[bib9] Bini L.M., Coelho A.S.G., Diniz-Filho J.A.F. (2001). Is the relationship between population density and body size consistent across independent studies? A meta-analytical approach. Rev. Bras. Biol..

[bib11] Blake Judith (1989). Family Size and Achievement.

[bib12] Bleakley Hoyt (2007). Disease and development: evidence from hookwork eradication in the American South. Q. J. Econ..

[bib13] Bozzoli Carlos, Deaton Angus, Quintana-Domeque Climent (2009). Adult height and childhood disease. Demography.

[bib14] Cameron Lisa, Shah Manisha, Olivia Susa (2013). Impact Evaluation of a Large-scale Rural Sanitation Project in Indonesia.

[bib15] Case Anne, Paxson Christina (2008). Stature and status: height, ability, and labor market outcomes. J. Polit. Econ..

[bib16] Checkley William, Buckley Gillian, Gilman Robert H., Assis Ana MO., Guerrant Richard L., Morris Saul S., Mølbak Kåre, Valentiner-Branth Palle, Lanata Claudio F., Robert E Black, The Childhood Malnutrition and Infection Network (2008). Multi-country analysis of the effects of diarrhoea on childhood stunting. Int. J. Epidemiol..

[bib17] Chen Alice, Oster Emily, Williams Heidi (2016). Why is infant mortality higher in the United States than in Europe?. Am. Econ. J. Econ. Pol..

[bib18] Clasen T., Boisson S, Routray P., Torondel B., Bell M., Cumming O., Ensink J., Freeman M., Jenkins M., Odagiri M., Ray S. (2014). Effectiveness of a rural sanitation programme on diarrhoea, soil-transmitted helminth infection, and child malnutrition in Odisha, India: a cluster-randomised trial. Lancet Global Health.

[bib19] Coffey Diane (2015). Prepregnancy body mass and weight gain during pregnancy in India and sub-Saharan Africa. Proc. Natl. Acad. Sci. Unit. States Am..

[bib20] Coffey Diane, Hathi Payal (2016). Underweight and pregnant: designing universal maternity entitlements to improve health. Indian J. Human Develop..

[bib21] Coffey Diane, Spears Dean (2017). Where India Goes: Abandoned Toilets, Stunted Development, and the Costs of Caste.

[bib22] Coffey Diane, Spears Dean (2018). Implications of WASH Benefits trials for water and sanitation. Lancet Global Health.

[bib23] Coffey Diane, Spears Dean (2018). Neonatal Death in India: the Effect of Birth Order in a Context of Maternal Undernutrition.

[bib24] Coffey Diane, Spears Dean (2018). Open defecation in rural India, 2015-16: levels and trends in NFHS4. Econ. Polit. Wkly..

[bib25] Coffey Diane, Geruso Michael, Dean Spears (2018). Sanitation, disease externalities and Anaemia: evidence from Nepal. Econ. J..

[bib26] Coffey Diane, Gupta Aashish, Hathi Payal, Khurana Nidhi, Dean Spears, Srivastav Nikhil, Vyas Sangita (2014). Revealed preference for open defecation. Econ. Polit. Wkly..

[bib27] Coffey Diane, Gupta Aashish, Hathi Payal, Dean Spears, Srivastav Nikhil, Vyas Sangita (2017). Understanding open defecation in rural India: untouchability, pollution, and latrine pits. Econ. Polit. Wkly..

[bib28] Coffey Diane, Khera Reetika, Dean Spears (2017). Intergenerational Effects of Women's Status: Evidence from Child Height in Joint Indian Households.

[bib29] Cunha Flavio, James J Heckman, Schennach Susanne M. (2010). Estimating the technology of cognitive and noncognitive skill formation. Econometrica.

[bib30] Currie Janet, Vogl Tom (2013). Early-life health and adult circumstance in developing countries. Ann. Rev. Econ..

[bib31] Cutler David, Miller Grant (2005). The role of public health improvements in health advances: the twentieth-century United States. Demography.

[bib32] Das Gupta Monica (1995). “Perspectives on Women's autonomy and health outcomes. Am. Anthropol..

[bib33] Deaton Angus (2007). Height, health and development. Proc. Natl. Acad. Sci. Unit. States Am..

[bib34] Deaton Angus (2013). The Great Escape: Health, Wealth, and the Origins of Inequality.

[bib35] DiNardo John, Fortin Nicole M., Lemieux Thomas (1996). Labor market institutions and the distribution of wages, 1973-1992: a semiparametric approach. Econometrica.

[bib36] Drèze Jean, Sen Amartya (2013). An Uncertain Glory: India and its Contradictions.

[bib38] Duh Josephine, Spears Dean (2017). Health and hunger: disease, energy needs, and the Indian calorie consumption puzzle. Econ. J..

[bib40] Galiani Sebastian, Paul Gertler, Schargrodsky Ernesto (2005). Water for life: the impact of the privatization of water services on child mortality. J. Polit. Econ..

[bib41] Gertler Paul, Heckman James, Pinto Rodrigo, Zanolini Arianna, Vermeersch Christel, Walker Susan, Chang Susan M., Grantham-McGregor Sally (2014). Labor market returns to an early childhood stimulation intervention in Jamaica. Science.

[bib42] Gertler Paul, Shah Manisha, Alzua Maria Laura, Cameron Lisa, Martinez Sebastian, Patil Sumeet (2015). How Does Health Promotion Work? Evidence from the Dirty Business of Eliminating Open Defecation.

[bib43] Geruso Michael (2012). Black-white disparities in life expectancy: how much can the standard SES variables explain?. Demography.

[bib44] Geruso Michael, Spears Dean (2018). Neighborhood sanitation and infant mortality. Am. Econ. J. Appl. Econ..

[bib45] Ghosh Arabinda, Gupta Aashish, Spears Dean (2014). Are children in West Bengal shorter than children in Bangladesh?. Econ. Polit. Wkly..

[bib46] Guerrant R.L., Schorling J.B., McAuliffe J.F., MA de Souza (1992). Diarrhea as a cause and an effect of malnutrition: diarrhea prevents catch-up growth and malnutrition increases diarrhea frequency and duration. Am. J. Trop. Med. Hyg..

[bib47] Hammer Jeffrey, Spears Dean (2016). Village sanitation and child health: effects and external validity in a randomized field experiment in rural India. J. Health Econ..

[bib48] Han Zhen, Lutsiv Olha, Mulla Sohail, Rosen Allison, Joseph Beyene, McDonald Sarah D. (2011). Low gestational weight gain and the risk of preterm birth and low birthweight: a systematic review and meta-analyses. Acta Obstet. Gynecol. Scand..

[bib49] Hathi Payal, Haque Sabrina, Pant Lovey, Coffey Diane, Dean Spears (2017). Place and child health: the interaction of population density and sanitation in developing countries. Demography.

[bib50] Hatton Timothy (2013). How have Europeans grown so tall?.

[bib51] Headey Derek (2015). The nutritional impacts of sanitation at scale: Ethiopia, 2000-2011. Neudc Conference Paper.

[bib52] Headey Derek, Hoddinott John, Ali Disha, Roman Tesfaye, Dereje Mekdim (2015). The other Asian enigma: explaining the rapid reduction of undernutrition in Bangladesh. World Dev..

[bib53] Hedges Larry V., Olkin Ingram (1985). Statistical Method for Meta-analysis.

[bib54] Hoddinott John, Alderman Harold, Jere R Behrman, Haddad Lawrence, Horton Susan (2013). The economic rationale for investing in stunting reduction. Matern. Child Nutr..

[bib55] Humphrey Jean H. (2009). Child undernutrition, tropical enteropathy, toilets, and handwashing. Lancet.

[bib57] Institute of Medicine (1990). Nutrition during Pregnancy: Part I, Weight Gain.

[bib58] Jayachandran Seema, Pande Rohini (2013). Why Are Indian Children Shorter than African Children?.

[bib59] Jayachandran Seema, Pande Rohini (2017). Why are Indian children so short? The role of birth order and son preference. Am. Econ. Rev..

[bib60] Jensen Robert (2012). Another mouth to feed? The effects of (In)Fertility on malnutrition. CESifo Econ. Stud..

[bib61] Kassebaum Nicholas J., Jasrasaria Rashmi, Naghavi Mohsen, Sarah K Wulf, Johns Nicole, Lozano Rafael, Regan Mathilda, Weatherall David, Chou David P., Eisele Thomas P. (2014). A systematic analysis of global anemia burden from 1990 to 2010. Blood.

[bib62] Kohler Hans-Peter (2012). Challenge Paper on “Population Growth”.

[bib63] Kosek Margaret, the MAL-ED network (2013). Fecal markers of intestinal inflammation and permeability associated with the subsequent acquisition of linear growth deficits in infants. Am. J. Trop. Med. Hyg..

[bib65] Lin Audrie, Arnold Benjamin F., Afreen Sadia, Goto Rie, Huda Tarique Mohammad Nurul, Haque Rashidul, Raqib Rubhana, Unicomb Leanne, Ahmed Tahmeed, Colford, John M., Luby Stephen P. (2013). Household environmental conditions are associated with enteropathy and impaired growth in rural Bangladesh. Am. J. Trop. Med. Hyg..

[bib66] Maluccio John A., Hoddinott John, Jere R Behrman, Martorell Reynaldo, Agnes R Quisumbing, Aryeh D Stein (2009). The impact of improving nutrition during early childhood on education among Guatemalan adults. Econ. J..

[bib67] Padhi Bijaya K., Kelly K Baker, Dutta Ambarish, Oliver Cumming, Freeman Matthew C., Satpathy Radhanatha, Das Bhabani S., Panigrahi Pinaki (2015). Risk of adverse pregnancy outcomes among women practicing poor sanitation in rural India: a population-based prospective cohort study. PLoS Med..

[bib68] Panagariya Arvind (2013). Does India really suffer from worse child malnutrition than sub-saharan Africa?. Econ. Polit. Wkly..

[bib69] Patil Sumeet R., Arnold Benjamin F., L Salvatore Alicia, Briceno Bertha, Ganguly Sandipan, Colford John M., Gertler Paul J. (2014). “The effect of India's total sanitation campaign on defecation behaviors and child health in rural Madhya Pradesh: a cluster randomized controlled trial. PLoS Med..

[bib70] Pickering Amy J., Djebbari Habiba, Lopez Carolina, Coulibaly Massa, Alzua Maria Laura (2015). Effect of a community-led sanitation intervention on child diarrhoea and child growth in rural Mali: a cluster-randomised controlled trial. Lancet Global Health.

[bib71] Prendergast Andrew J., Rukobo Sandra, Chasekwa Bernard, Mutasa Kuda, Ntozini Robert, Mbuya Mduduzi N.N., Jones Andrew, Moulton Lawrence H., Stoltzfus Rebecca J., Humphrey Jean H. (2014). Stunting is characterized by chronic inflammation in Zimbabwean infants. PLoS One.

[bib73] Ramalingaswami Vulimiri, Urban Jonsson, Rohde Jon (1996). “Commentary: the Asian Enigma.” the Progress of Nations.

[bib75] Rutstein Shea Oscar, Rojas Guillermo (2006). Guide to DHS Statistics.

[bib77] Smith Michelle I., Yatsunenko Tanya, Manary Mark J., Trehan Indi, Mkakosya Rajhab, Cheng Jiye, Kau Andrew L., Rich Stephen S., Concannon Patrick, Mychalecky Josyf C., Liua Jie, Houpt Eric, Li Jia V., Holmes Elaine, Nicholson Jeremy, Knights Dan, Ursell Luke K., Knight Rob, Gordon Jeffrey I. (2013). Gut microbiomes of malawian twin pairs discordant for kwashiorkor. Science.

[bib78] Solon Gary, Haider Steven J., Wooldridge Jeffrey (2013). What Are We Weighting for?.

[bib79] Spears Dean (2013). “How Much International Variation in Child Height Can Sanitation Explain?.

[bib80] Spears Dean, Haddad Lawrence (2015). The Power of WASH: Why Sanitation Matters for Nutrition.

[bib81] Spears Dean, Lamba Sneha (2016). Effects of Early-Life Exposure to Sanitation on Childhood Cognitive Skills. J. Hum. Resour..

[bib82] Spears Dean, Thorat Amit (2017). Caste, purity, and pollution and the puzzle of open defecation in India: evidence from a novel measure in a nationally-representative survey. Econ. Dev. Cult. Change.

[bib83] Spears Dean, Coffey Diane, Behrman Jere (2018). Understanding Child Height, Birth Order, and Fertility in India.

[bib85] Steckel Richard (2009). Heights and human welfare: recent developments and new directions. Explor. Econ. Hist..

[bib86] Tarozzi Alessandro (2008). Growth reference charts and the nutritional status of Indian children. Econ. Hum. Biol..

[bib87] Victora Cesar Gomes, de Onis Mercedes, Pedro Curi Hallal, Blössner Monika, Shrimpton Roger (2010). Worldwide Timing of Growth Faltering: Revisiting Implications for Interventions.

[bib88] Vyas Sangita, Kov Phyrum, Smets Susanna, Dean Spears (2016). “Disease externalities and net nutrition: evidence from changes in sanitation and child height in Cambodia, 2005–2010. Econ. Hum. Biol..

[bib89] Yaktine A., Rasmussen K. (2009). Weight Gain during Pregnancy: Reexamining the Guidelines.

