# Supplementary material for: Exposure to open defecation can account for the Indian enigma of child height
Source: J Dev Econ. 2020 Sep;146:102277. doi: 10.1016/j.jdeveco.2018.08.003 (PMC7457703; doi:10.1016/j.jdeveco.2018.08.003)
Supplement: Multimedia component 1 [file mmc1.pdf]

A Other related estimates in the literature

Section 6 integrated estimates of the effect of local open defecation on child height. Other recent studies of the health consequences of sanitation are generally consistent with these results. The studies in this section, unlike most of the studies in 6, do not specifically provide an estimate of an association between open defecation externalities and child height-for-age.

Hammer and Spears (2016) report results of a randomized, controlled trial of a sanitation program intended to reduce open defecation, conducted in a district of rural Maharashtra in 2004. Although the data do not measure open defecation behavior, so an IV estimate analogous to the Gertler, et al. meta-coefficient is not computed, the data do show evidence of an effect of the sanitation promotion program on average child height for age.[20]

A range of recent cross-sectional observational studies have found an association between a measure of open defecation and a measure of child height. Using DHS data from many countries, Fink et al. (2011) document an association between improved sanitation and the odds that a child is stunted, a binary indicator of being very short.[21] Spears et al. (2013) document a robust correlation between average open defecation and the fraction of children stunted, across Indian districts. Rah et al. (2015) find similar results by pooling data from three recent surveys of rural India.

Three new observational studies show effects of open defecation on other children's health outcomes that are known in the literature to be related to child height for age. Duflo et al. (2015) exploit variation in the timing of the turning-on of a village water and sanitation program to document a sharp effect on diarrhea, one important mechanism by which poor sanitation could lead to poor nutritional outcomes.[22] Geruso and Spears (2017) exploit a difference in sanitation behavior between Muslims and Hindus living in India to estimate externality effects of neighbors' open defecation on infant mortality; disease determinants of infant mortality are well-documented in the demographic and economic history literatures

[20]The analysis of the field experiment also critically assesses its external validity: because facts about how the 2004 research team and Maharashtra government selected the study district have been recorded, the authors can show that the experiment was conducted in a district that was unusually conducive to finding an effect, rather than in the less favorable districts that were originally considered.

[21]See also Smith and Haddad (2015) for a related macro-level analysis that similarly finds an important role for sanitation in explaining stunting.

[22]Also, Kumar and Vollmer (2013) use a propensity score matching analysis of Indian survey data to show an association between open defecation and survey-reported diarrhea.

to also shape population average height (Bozzoli et al., 2009; Hatton, 2013). Coffey et al. (2017) show that sub-regions of Nepal where open defecation has improved more quickly after a government sanitation program have also seen more rapid improvements in anemia, on average, a nutritional consequence that could plausibly be an effect of sanitation-related intestinal disease. Collectively, these new studies suggest that it is plausible that open defecation in India could have a large effect on average early-life net nutrition, the key determinant of average child height.

Finally, two studies have recently been discussed at conferences but not yet published. Reese et al. (2017) describe a matched-cohort study in Orissa, India that is designed to assess the effectiveness of a combined piped water and sanitation intervention by Gram Vikas. Preliminary findings in Heather Reese's dissertation show an improvement in child height.[23] Humphrey et al. (2015) describe the carefully-conducted SHINE trial of sanitation and nutrition, which is located in rural Zimbabwe. Population density in rural Zimbabwe is much lower than in rural India: Hathi et al.'s (2017) analysis of all GIS-coded DHS surveys predicts that open defecation would not be steeply associated with child height in sub-Saharan African contexts where population density is low. In the supplementary appendix, figure A.2 shows, using Zimbabwe's 2015 DHS, that this prediction is borne out in the data: in rural Zimbabwe, child height-for-age is essentially uncorrelated with the same type of PSU open defecation variable that is constructed for this paper's main analysis.

B Non-parametric reweighting decompositon

This section presents further details on the child-level results from section 5 of the paper.

B.1 Open defecation interacts with population density to predict height

The child-level results in section 4 of the paper use the log of open defecation density as the independent variable. For reference, table A.1 presents results using PSU open defecation: the fraction 0-1 of households in a child local primary sampling unit reporting defecating in the open. This table verifies the interaction between open defecation and population density that informs the paper's preferred specification.

[23]Reese, Heather (2017) Effectiveness of a combined sanitation and household-level piped water intervention on infrastructure coverage, availability and use, environmental fecal contamination, and child health in rural Odisha, India: a matched cohort study. PhD Dissertation. Rollins School of Public Health, Emory University.

Recall that the PSU fraction of households defecating in the open is the explanatory variable studied in the review of the literature in section 4 of the paper and in table 1. For open defecation, measured this way, to account for the India-Africa height gap would require the average coefficient within India to be -0.54 or -0.47 height-for-age standard deviations for a 0 to 1 difference in open defecation. Gertler, *et al.* (2015) estimate an instrumented coefficient of -0.46, using results from three randomized experiments.

Section 4 uses the same pooled DHS dataset as Jayachandran and Pande (2013, 2017). They report an open defecation coefficient of -0.358, pooling data from India and Africa and using a binary indicator of household open defecation. There are two reasons to believe that this is an underestimate of the relevant parameter, which is the extent to which the height of the average Indian child would increase if counterfactually exposed to African open defecation. First, this household-level measure ignores externalities of open defecation, capturing only differences across households. Second, this estimate pools the large Indian effect of interest with the smaller effect in Africa, where population density is lower.

Table A.1 confirms both of these issues. Column 1 finds a larger coefficient of -0.438 when PSU open defecation is used instead of household open defecation, pooling Indian and African data. Columns 2 and 3 verify that the gradient between open defecation and height is steeper in India than in Africa. In a result designed to be comparable to the estimates in the literature in table 1 of the paper, column 4 estimates that children in India in localities where every household reports defecating in the open are 0.557 height-for-age standard deviations shorter than children in India where no households report defecating in the open, after controlling for their mothers' height in centimeters and a set of asset ownership indicators interacted with an indicator for urban residence.

Finally, columns 5 and 6 verify the statistical significance of the interaction between open defecation and population density, which informs our preferred specification's use of the density of open defecation as an explanatory variable. Recall that population density itself does not explain essentially any of the India-Africa height gap and that section 3 of the paper confirmed that population density itself is not correlated with DHS-average child height.

B.2 Summary statistics by birth order and sibsize

Table A.2 presents summary statistics for these data. Demographic categories are correlated with child height, especially in India. In particular, Jayachandran and Pande (2017) have recently emphasized a correlation between child birth order and height: they observe that birth order has a more steeply negative gradient with child height in India than in Africa.

Table A.2 is structured by child birth order and mother's fertility[24] in order to permit the reader to visualize separately the correlates of a child's birth order and of her mother's fertility, that is to say, of the size of her siblingship at the time of the DHS survey, when height is measured.

A well developed literature in economic demography emphasizes the importance of separately controlling for sibsize and birth order when trying to identify the effects of either; they are mechanically correlated because high birth order children must come from high fertility mothers (Blake, 1989; Black et al., 2015). These data requirements matter here because the Indian DHS only measures the height of children under 5, and some African DHS surveys do not even measure height up to age 5. One consequence of this is that only the youngest children in each family have height data: 97% of children with measured height in the DHS are either the most recent or second most recent of their mother's births. This means that child birth order and the size of a mother's fertility are particularly strongly correlated in the sub-sample of children in the DHS with measured height: the correlation between a child's birth order and the number of children born to her mother is 0.64 among all children observed in the DHS birth history, but is 0.96 in the sub-sample with measured height.

Despite this correlation, the large size of the dataset permits some scope to distinguish between the role of mother's fertility (that is, the size of the child's siblingship) and the role of the child's birth order. Table A.2 presents means for each observed combination of mother's fertility (in rows) and child birth order (in columns) for each of four variables: child height-for-age,[25] an indicator for the mother's literacy, the mother's height in centimeters, the fraction of households in the child's local PSU who report defecating in the open, and our constructed measure of open defecation density.

Comparisons *down rows* show that higher fertility is considerably more correlated with disadvantage in India than in Africa. For an example from the top corner of the table, in India first-born children in siblingships of 2 are about 0.09 standard deviations shorter than first-borns in siblingships of 1; in Africa first-born children from siblingships of 2 are about 0.02 standard deviations *taller* than first-borns from siblingships of 1. Mother's literacy in India declines by almost 40 percentage points from singletons to those with 4 children

[24]In the table, we follow the DHS documentation and convention in demography by calling this variable "children ever born."

[25]The table presents residuals after height-for-age is regressed on 119 age-in-months by sex dummies. Age-in-months by sex is the level at which the WHO height reference charts are constructed; therefore, these indicators control for any bias introduced by any discrepancy between average height in our sample and in the reference sample. Child height-for-age is well known to be declining in the first two years of life in poor countries where stunting is common (Victora et al., 2010); figure 1 shows that the average child in the DHS with measured height is older in India than in Africa – because early-life mortality is greater in Africa – so these controls for age are necessary.

(Panel B) and height declines by about a centimeter (Panel C); in Africa over the same range mother's literacy declines by less than 20 percentage points and mother's height *increases*. Similarly, children born to higher-fertility mothers are exposed to much more open defecation in India than children exposed to lower-fertility mothers, by a steeper gradient than in Africa.

In contrast, comparisons *across columns* show that, at the same level of mother's fertility, later born children in India do not appear more disadvantaged than later born children in Africa. Panel A considers our variable of interest, child height-for-age. In all cases, compared adjacently in rows, later-born children of the same siblingship size are taller than earlier-born children. In India, this apparent advantage is much larger than in Africa for siblingships of size 3 or 4: for example, Indian third-borns are 0.12 standard deviations taller than Indian second-borns, both of mothers to which 3 children have been born; African third-borns are only 0.01 standard deviations taller. In general, in Africa – where fertility is higher, on average, and high fertility is less of a marker of disadvanage – there is not much difference by fertility or birth order: no average differs by as much as 0.04 standard deviations from the average for singleton first-borns. In India, however, there is a steep gradient in mother's fertility. Spears, Coffey, and Behrman (2018) document that empirical strategies, such as mother fixed effects, that succeed in other studies in the birth order literature are unable to convincingly separate sibsize from birth order in the DHS height subsample, due to the facts that the DHS only measures the youngest children and that age, height-for-age, and birth order are correlated.

Later birth order children may appear to be relatively disadvantaged in India because high fertility is more negatively selective there. As a result, our decomposition analysis takes care to allow for different consequences of fertility between India and Africa – both by controlling for the mean difference in fertility and by computing projections within demographic subsamples in case of any interactions – as well as any role of birth order.

B.3 Robustness to changes in the African sample

Figure A.1 is included to verify that our result is not driven by the particular sample of sub-Saharan African DHS country-years included, in which we follow the sample constructed by Jayachandran and Pande (2013, 2017). The figure presents decomposition results as the increase in Indian child height associated with matching African height, as a fraction of the 0.146 standard deviation height-for-age gap. The figure plots a histogram of the distribution of results from 1,000 Monte Carlo replications, in each of which each African DHS had an independent 25% chance of being dropped from the sample.

Figure A.1: Robustness of decomposition result to changes in the African sample

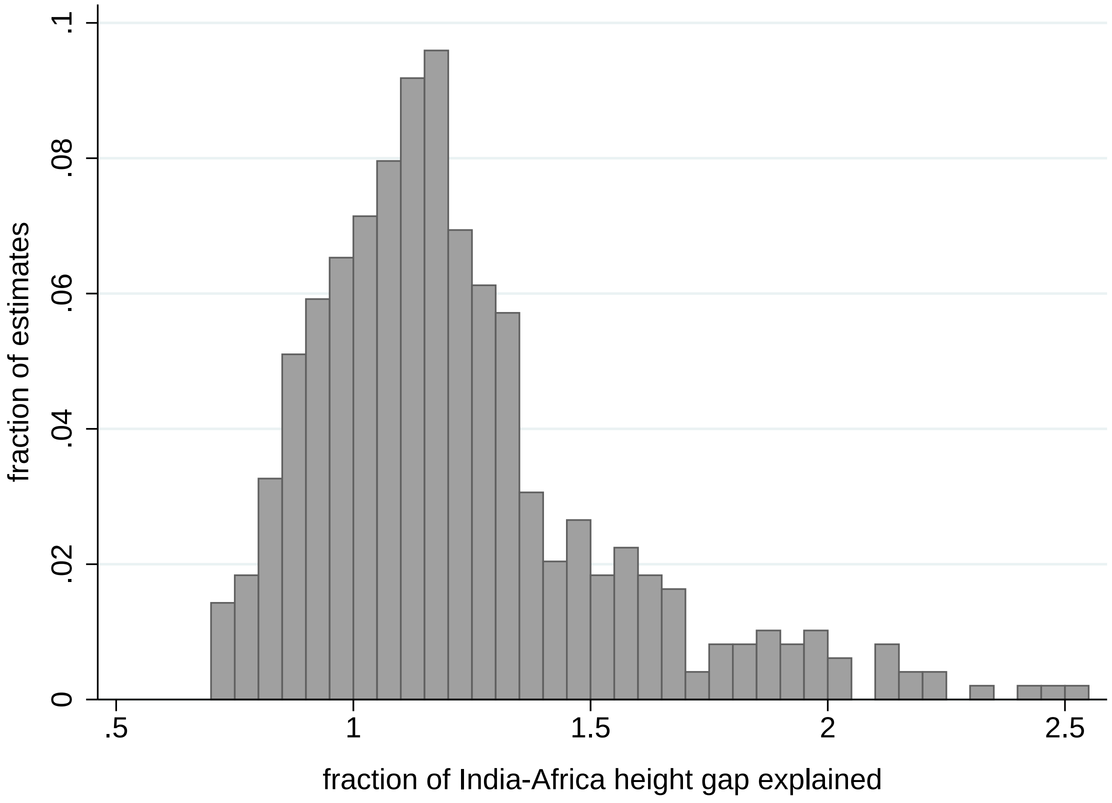

Note: Child-level sample. Each observation is the fraction of a random sub-sample's height gap linearly explained by the log of the density of open defecation; each sample has an i.i.d. 25% chance of dropping each African DHS survey.

Figure A.2: Local open defecation does not predict child height-for-age in demographic data from rural Zimababwe

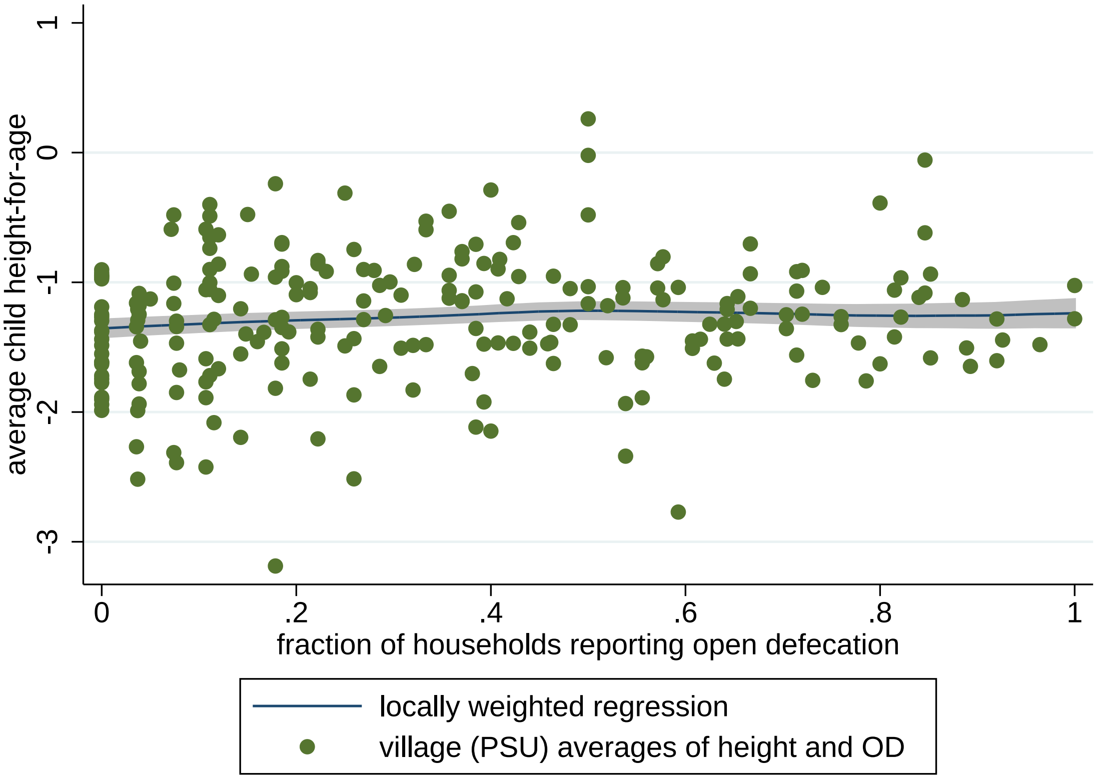

Note: Computed from Zimbabwe's 2015 DHS; rural observations only. The line is a locally weighted regression with a 95% confidence interval. Dots represent PSU (village) averages. The horizontal axis is the fraction of households in the household recode that report open defecation, among sampled households in the child's PSU.

Table A.1: Local open defecation and child height, India and Africa

	(1)	(2)	(3)	(4)	(5)	(6)
sample:	full	Africa	India	India	full	full
PSU open defecation	-0.438***	-0.305***	-0.730***	-0.557***	-0.306***	-0.301***
	(0.0186)	(0.0233)	(0.0299)	(0.0355)	(0.0233)	(0.0233)
India					0.149***	0.167***
					(0.0213)	(0.0221)
India × PSU OD					-0.423***	-0.396***
					(0.0379)	(0.0420)
population density						-0.0000269**
						(0.00000863)
density × PSU OD						-0.0000868*
						(0.0000397)
mother's height				0.0525***		
				(0.00161)		
asset indicators × urban				✓		
age-in-months × sex	✓	✓	✓	✓	✓	✓
n (children under 5)	170,149	127,668	42,481	42,231	170,149	170,149

Standard errors clustered by survey PSU in parentheses. p-values: † $p < 0.1$, * $p < 0.05$, ** $p < 0.01$, *** $p < 0.001$. Open defecation is a fraction 0 to 1.

Table A.2: Summary statistics by demographic category: Indian and African children

	India				Africa			
**Panel A: Child height for age z-score, residuals after age-in-months by sex dummies**								
	1st born	2nd born	3rd born	4th born	1st born	2nd born	3rd born	4th born
1 child ever born	0.178				0.039			
2 children ever born	0.089	0.091			0.061	0.083		
3 children ever born		-0.233	-0.113			0.041	0.052	
4 children ever born			-0.428	-0.192			-0.010	0.046
**Panel B: Mother's literacy**								
	1st born	2nd born	3rd born	4th born	1st born	2nd born	3rd born	4th born
1 child ever born	0.785				0.746			
2 children ever born	0.731	0.745			0.667	0.696		
3 children ever born		0.568	0.581			0.614	0.648	
4 children ever born			0.417	0.421			0.562	0.601
**Panel C: Mother's height**								
	1st born	2nd born	3rd born	4th born	1st born	2nd born	3rd born	4th born
1 child ever born	152.3				157.8			
2 children ever born	152.2	152.3			157.8	157.9		
3 children ever born		151.8	151.8			158.0	158.1	
4 children ever born			151.5	151.4			158.2	158.3
**Panel D: PSU open defecation**								
	1st born	2nd born	3rd born	4th born	1st born	2nd born	3rd born	4th born
1 child ever born	0.367				0.287			
2 children ever born	0.437	0.392			0.310	0.292		
3 children ever born		0.501	0.498			0.313	0.302	
4 children ever born			0.529	0.510			0.332	0.323
**Panel E: Open defecation density**								
	1st born	2nd born	3rd born	4th born	1st born	2nd born	3rd born	4th born
1 child ever born	3.21				1.75			
2 children ever born	3.55	3.34			1.92	1.78		
3 children ever born		4.05	3.81			1.98	1.86	
4 children ever born			4.11	4.01			2.02	1.96

Note: Child level sample. Only the last two births of each mother are plotted because 97% of children in the data are either the most recent or second-most-recent birth, because the DHS only measures height of children under 5 years old. Panel A plots averages of residuals after child-level height-for-age z scores are regressed on 120 age-in-months by sex indicators.

Table A.3: Controls for age: consequences and alternatives

	(1)	(2)	(3)	(4)	(5)	(6)
ages included:	0-59	0-59	24-59	24-59	0-59	0-59
India	-0.146***	0.0167	-0.0818***	0.0893***	-0.0882***	0.0783***
	(0.0156)	(0.0163)	(0.0174)	(0.0182)	(0.0156)	(0.0162)
ln(OD density)		-0.0922***		-0.0990***		-0.0944***
		(0.00376)		(0.00423)		(0.00375)
age-in-months FEs					✓	✓
n (children under 5)	170,149	170,149	97,955	97,955	170,149	170,149

Note: Standard errors clustered by survey PSU in parentheses. p-values: † $p < 0.1$, * $p < 0.05$, ** $p < 0.01$, *** $p < 0.001$.
Observations: This table implements the OLS-as-decomposition regression of equation 3 with two different ways of "controlling" for the correlation between child age and child height-for-age: columns 5 and 6 control for 59 age-in-months fixed effects, while columns 3 and 4 simply omit children under 2 years old. This is important because height-for-age falls in age over the first two years of life, on average, and children with measured height in the DHS in India are older, on average, than children in sub-Saharan Africa (see figure 1), due to differences in mortality rates.
Three conclusions are visible in the results:

- Comparing column 1 with columns 3 and 5, controlling for age changes the amount of the "Asian Enigma" gap to be explained, although a substantial difference remains. Part of the reason Indian children appear shorter is because they are older, and have had more time to accumulate height shortfalls.
- Comparing columns 3 and 4 against columns 5 and 6, it appears that these two different methods of "controlling" for age produce very similar results.
- Comparing columns 2, 4, and 6, the coefficient on the density of open defecation is not sensitive to either of these age controls.

One question is whether mortality selection might bias the estimate of the association between open defecation and child height, if children who would be unusually short are the ones who are likely to die from exposure to open defecation. However, an analysis of historical infant mortality rates (from times when early life mortality was more common) by Bozzoli et al. (2009) finds that the required mortality rate for such selection to be quantitatively important is very large.

We confirm this in our sample with the following procedure. 4% of the births reported in the five years before the survey do not have measured height; these are children who would have been eligible to have their height measured if they survived. We hypothetically assign each of these the tenth-percentile height among measured children who share their age-in-months and sub-national region (such as Uttar Pradesh); thus, we assume that they would have been unusually short, if measured. When we re-estimate column 2 with these 177,261 real and counterfactual observations, we find a coefficient on open defecation density of -0.099, which is very similar to the -0.092 observed on the real sample of 170,149.

I thank Jere Behrman and Subha Mani for suggestions that led to this table.

Table A.4: Reweighting robustness check: Results are robust to using DHS sample weights

covariates also reweighted over					
sex	sibsize	assets	urban	counterfactual increase	loss of SSA sample
				0.20	0%
✓				0.21	0%
	✓			0.20	0%
✓	✓			0.20	0%
✓	✓	✓		0.14	1.2%
✓	✓	✓	✓	0.17	3.6%

Note: Compare with the first column (full sample) of main result table 2, which does not use DHS sampling weights, but rather weights each observation equally.

Table A.5: Indian children reweighted to match African sample on open defecation and other properties (all ages, by sex)

sex	sibsize at survey	birth order	assets	urban	mom literate	dad any education	mom age at birth	males	females
								0.200	0.227
✓								0.200	0.227
	✓							0.211	0.246
✓	✓							0.211	0.246
✓	✓		✓					0.198	0.207
✓	✓		✓	✓				0.220	0.239
✓	✓		✓	✓	✓			0.186	0.200
✓	✓		✓	✓	✓	✓		0.174	0.214
✓	✓		✓	✓	✓	✓	✓	0.143	0.198
		✓						0.220	0.241
✓		✓						0.220	0.241
✓		✓	✓					0.199	0.212
✓		✓	✓	✓				0.210	0.231
✓		✓	✓	✓	✓			0.179	0.190
✓		✓	✓	✓	✓	✓		0.163	0.202
✓		✓	✓	✓	✓	✓	✓	0.141	0.191
✓	✓	✓	✓	✓	✓	✓	✓	0.141	0.228
			✓					0.192	0.212
			✓	✓				0.204	0.213
			✓	✓	✓			0.164	0.187
			✓	✓	✓	✓		0.154	0.175
			✓	✓	✓	✓	✓	0.163	0.166

Note: Columns correspond to sub-samples. Rows correspond to sets of other properties. Differences presented are the difference between the height of Indian children reweighted to match African children on that row's other properties and the height of Indian children reweighted to match the African joint distribution of open defecation and the same other properties. Although it makes no difference in these samples, rows corresponding to adding a sex control are included for comparability with the main results tables.
